# Adequacy of Standard Models for Long-Term Behavior of Lightweight Concrete with Sintered Aggregate Under Cyclic Loading

**DOI:** 10.3390/ma19010059

**Published:** 2025-12-23

**Authors:** Paweł M. Lewiński, Zbigniew Fedorczyk, Przemysław Więch, Łukasz Zacharski

**Affiliations:** Building Research Institute (ITB), Filtrowa 1, 00-611 Warsaw, Poland; z.fedorczyk@itb.pl (Z.F.); p.wiech@itb.pl (P.W.); l.zacharski@itb.pl (Ł.Z.)

**Keywords:** lightweight aggregate concrete, long-term tests, shrinkage, creep, long-term concrete deformation models

## Abstract

This paper presents an experimental determination of the long-term mechanical properties of lightweight concrete with sintered aggregate under cyclic loading and the corresponding analytical standard models. The research was designed around two concrete mixtures. Multiple tests were conducted at the Building Structures, Geotechnics and Concrete Laboratory of the Building Research Institute (ITB), using various equipment including creep-testing machines and tensometric measurements of sample deformations. As a result of these tests, in addition to strength properties, the following time-dependent parameters were determined: the secant modulus of elasticity, shrinkage strains, and creep-recovery strains under cyclic loading. For the parameterization and modeling of constitutive equations, an analysis of creep strains under cyclic loads was carried out, taking into account the integral hereditary law according to the Boltzmann superposition principle and the long-term models formulated according to the following standards and pre-standards: Eurocode 2 (2004), Model Code 2010, Model Code 2020, and Eurocode 2 (2023). The results from the individual models were compared with the test results using the rules for evaluating correction factors for models determined according to Eurocode 2 (2023). It was concluded that the development of creep strain is correctly modeled by the aforementioned standard methods, albeit with the aforementioned correction factors. One of the research objectives was to determine whether the ratchetting phenomenon could be observed during creep of the tested concrete under cyclic loading; however, due to the very low level of plastic deformation, this phenomenon was not detected. The research confirmed the suitability of lightweight concrete with sintered aggregate for use in cyclically loaded concrete structures.

## 1. Introduction

### 1.1. Development of Research on Standard Models

The long-term inelastic deformations of concrete under cyclic loading have been the subject of publications by many well-known authors. This paper also describes such phenomena, but represents a further step forward compared to the authors’ earlier research, by considering the effects of long-term cyclic loading on lightweight aggregate concrete samples. Mathematical models describing these phenomena were developed to provide a validated algorithm for predicting the long-term deformation of such concrete under time-varying loads. The behavior of lightweight concrete made with a relatively new sintered aggregate, the study of which is presented in this paper, was previously described in the authors’ publication [1]. The aim of that publication was to determine the short-term and long-term mechanical properties of lightweight aggregate concrete, but within an earlier research stage that did not include the cyclic phenomena discussed here and their mathematical modeling. Whaley and Neville [2] demonstrated that, compared to static stress, cyclic loading accelerates the inelastic deformation of concrete. The creep of plain and structural concrete is addressed in the fundamental monographs by Neville et al. [3,4]. In work [5], the theoretical relationship that accurately describes static creep was extended and transformed so that cyclic creep can be related to static creep as a function of stress range and time. Bažant and Panula, through a series of multi-stage studies (see, e.g., [6,7]), used optimization techniques to fit the numerous test data available in the literature and proposed a practical model, the BP model, to predict the creep and shrinkage of concrete, taking into account concrete mix composition, strength, age of concrete at loading, environmental conditions, size, and shape, among other factors. These studies yielded more accurate results than, for example, the ACI or CEB-FIP models of the time, which contributed to the development of more accurate models in later design standards. Work [8] investigated the effect of the frequency of load intervals on stress relaxation. The increase in creep due to humidity cycling was partially formulated based on diffusion theory considerations. The increase in creep due to cyclic humidity changes was formulated in [9], partly based on diffusion theory. Paper [10] presents a computational procedure to predict the deflections of RC beams subjected to repeated loading. A semi-empirical constitutive BP model is adopted to account for the effects of creep (basic, drying, and cyclic) and shrinkage. Bažant and Murphy proposed the more advanced B3 model for the characterization of concrete creep and shrinkage [11]. Paper [12] outlines the experimental program and its results on the bond behavior of conventional reinforcing bars in lightweight aggregate concrete (LWAC) under cyclic loading. The cyclic creep theory of concrete based on the Paris law [13] is applicable to structures subjected to repeated variable loads over longer time intervals, leading to the growth of fatigue micro-cracks at subcritical loads. A comparative analysis of concrete behavior under compressive creep and cyclic loading [14] shows that cyclic deformations are significantly higher than creep strains despite similar mean stress levels; however, this publication also applies to high-frequency loads. Extensive information in this area is provided by the *fib* Model Code 2010 (MC 2010) [15] (see also [16]) and the *fib* Model Code 2020 (MC 2020) [17]. Under cyclic loads, the creep recovery phenomenon was observed by the authors, which is also accounted for by the models presented in this paper. Creep recovery and creep relaxation have been addressed in a number of important publications, including the papers by Bednář [18], Brooks [19], Cao et al. [20], Chen et al. [21], Counto [22], Li et al. [23], Mei et al. [24,25], Neville [26], Qu et al. [27], Yue and Taerwe [28], and Zhou et al. [29]. Residual strains after unloading were observed by Rossi et al. [30]. Huang et al. studied the creep of hydraulic concrete subjected to cyclic loading [31,32]. Comparative analyses of tensile and compressive creep of different concretes were addressed in a series of comprehensive articles by Hilaire et al. [33], Hui et al. [34], Ji et al. [35], Kim et al. [36], Klasusen et al. [37], Ranaivomanana et al. [38], Rossi et al. [39,40], and Wei et al. [41].

Shrinkage phenomena in LWAC, including shrinkage cracks, were analyzed in several published works, including [42,43,44]. Early thermal-shrinkage stresses resulting from hydration heat release during the setting process in LWAC were analyzed in [45,46,47]. Shrinkage and creep phenomena in LWAC were also addressed in works [48,49,50,51,52,53,54,55]. The creep phenomenon in lightweight aggregate concrete reinforced with steel fibers under tension was also investigated; see, e.g., [56]. Chia et al. [57] presented creep and shrinkage tests of a high-performance ultralightweight cement composite with a strength of 60 MPa. Cui et al. [58] demonstrated the effect of curing time on the creep of lightweight aggregate concrete. Lye et al. [59] proposed estimation models for the creep and shrinkage of concrete made with natural, recycled, and secondary aggregates. Singh et al. [60] experimentally determined the creep coefficients for sintered fly ash lightweight aggregate concrete and verified them against creep models.

In the work of Wojewódzki et al. [61], proposals for models addressing the relaxation function and the description of the triaxial stress state under creep conditions were presented, which were also recently included in MC 2020 [17]. The influence of various factors on the long-term properties of concrete has been analyzed in detail in a number of publications, including the monographs by Biliszczuk (see, e.g., [62,63]). Extensive and highly detailed reviews of constitutive models in the field of concrete rheology, together with their analysis, are included in the monographs by Mitzel [64] and the more recent one by Brunarski [65]. The works described above helped the formulation of successive standard models, i.e., the EC2:2004 [66] model and subsequently the pre-standard documents such as MC 2010 [15], MC2020 [17], and EC2:2023 [67], all of which are considered herein.

### 1.2. Long-Term Tests of Concrete on a New Type of Lightweight Sintered Aggregate

Lightweight concrete with sintered aggregate, due to its properties, has been widely used in construction, including in high-rise buildings, prefabricated structures, bridges, and drilling platforms [68]. Production of the *Pollytag* aggregate, characterized by the highest crushing resistance among the artificial aggregates used in Poland, ceased in 2015. That same year, a new lightweight sintered aggregate (LSA) called *Certyd*, produced using a slightly different technology, was introduced into design and construction practice. The following year, long-term testing of concrete made with this aggregate began, with the aim of using this concrete in the construction of post-tensioned concrete floors.

As a result of extensive research conducted in the IMiKB (Instytut Materiałów i Konstrukcji Budowlanych (Institute of Building Materials & Structures)) laboratory of the Cracow University of Technology, mainly by Domagała [69], Mieszczak [70,71,72,73,74], and Szydłowski [71,72,74,75,76,77], significant publications and monographs have been published on the long-term properties of concrete with lightweight sintered aggregate since 2016. These tests involved determining the mechanical properties of concrete, including compressive strength, tensile strength, and modulus of elasticity, as well as characterizing the development of concrete shrinkage and creep. The test results demonstrate that the tested concrete has properties that allow it to be used in prestressed structures. According to these works, a low creep coefficient was obtained for lightweight concrete with sintered aggregate. The papers by authors such as Szydłowski et al. [71,72,74,75,76,77] are of fundamental importance to the prospects of using lightweight sintered aggregate in the design of prestressed concrete structures. Seruga and Szydłowski [78] investigated the bond strength of lightweight aggregate concrete to plain seven-wire non-pretensioned steel strands. Szydłowski and Mieszczak [79] analyzed the use of lightweight aggregate concrete for constructing post-tensioned long-span slabs. The significant monograph [80] presents the state of the art in the field of lightweight structural concrete of the type considered.

A prerequisite for these new applications is a reliable study of the long-term properties of the concrete under consideration, based on current standards and guidelines. For this reason, the Building Research Institute (ITB) conducted research on a number of mechanical properties of lightweight concrete with sintered aggregate [81,82], using the same aggregate as that tested in the IMiKB laboratory of the Cracow University of Technology. The tests were conducted to determine the long-term properties of shrinkage and creep under cyclic loading at the Building Structures, Geotechnics and Concrete Laboratory of the ITB, in addition to the short-term strength properties of the lightweight concrete under consideration. In this paper, the development of creep and shrinkage of a normal-strength lightweight concrete of LC 45/50 class according to EC2:2004 [66] with two water-cement ratios, subjected to cyclic loading and unloading, is comparatively presented. A program was developed to test the mechanical properties of lightweight concrete with sintered aggregate under low-frequency cyclic loading.

The tests in the ITB strength testing laboratory were carried out on two lightweight concrete mixtures because, at the beginning of the research, the authors had only two creep testing machines at their disposal. With these devices, samples from only two concrete mixtures could be tested. A literature review was conducted to select the two concrete mixtures, ensuring the tests would be representative, both in terms of the testing of lightweight concrete itself and its applications in prestressed structures. As a result of the literature review, the concrete mixture compositions proposed by the LSA Białystok (Poland) company were adopted as pre-optimized formulations, the same as those used in the research conducted at the Cracow University of Technology [69,70,71,72,73,74,75,76,77,78,79]. Only two such mixture recipes were proposed by LSA, and they should therefore be considered representative. The method of selecting concrete mixtures and the important limitations regarding this selection are discussed in detail in Section 2.1.

## 2. Materials and Methods—Research Program of Long-Term Properties of Concrete with Sintered Aggregate

### 2.1. Preparation of Concrete Mixtures

The research involved determining the effect of cyclic loads on the long-term mechanical properties of lightweight concrete prepared from two types of concrete mixtures containing special sintered ceramic aggregate (Certyd, produced by LSA Białystok, Poland). Using the ingredients specified in Table 1 (see below), two concrete mixtures were prepared with water-cement ratios (W/C) of 0.4 and 0.5, respectively. The mixtures were prepared according to the LSA recommendations, as described in publication [77].

Using the ingredients listed in Table 1, two mixes were obtained, designated LC1 and LC2, which, as fresh concrete mixes, achieved densities of 1960 kg/m^3^ and 1980 kg/m^3^, respectively. After 28 days of curing, the dried concrete density was determined to be 1766 kg/m^3^ for the LC1 mix and 1777 kg/m^3^ for the LC2 mix in accordance with [83], yielding similar values to those reported in [77].

The admixtures BV 18 and SKY 686 (produced by Master Builders Solutions Polska, Myślenice, Poland) are a plasticizer end superplasticizer, respectively, manufactured in accordance with the reference documents for concrete admixtures. The primary raw materials of the BV 18 admixture are lignosulfonates, while those of the SKY 686 admixture are polycarboxylic ether. Both admixtures contain small amounts of chlorides (≤0.1% by weight) and alkalis (BV 18: ≤1.2% and SKY 686: ≤0.9% by weight).

### 2.2. Types of Tests and Corresponding Samples

The lightweight concrete with sintered aggregate was prepared in a concrete plant using a suitable mixer to ensure a homogeneous mix. After pouring the lightweight aggregate concrete into the prepared molds, forming the concrete blocks, extracting the drill cores, and curing under laboratory conditions, the following mechanical properties were tested in accordance with the appropriate procedures:

- Secant modulus of elasticity of concrete in accordance with [84];
- Compressive strength in accordance with [85];
- Axial tensile strength in accordance with [86];
- Tensile splitting strength in accordance with [87];
- Flexural strength in accordance with [88];
- Shrinkage strains in accordance with [89,90];
- Creep strains in accordance with [86].

For the tests of the secant modulus of elasticity [84], axial tensile strength, shrinkage in accordance with [90], and creep in accordance with [86], samples from boreholes with a nominal diameter of 94 mm and height of 190 mm (for compressive strength and elasticity modulus) or 282 mm (for creep and shrinkage) were used. For the remaining tests, cubic samples with dimensions of 150 × 150 × 150 mm or prismatic samples were prepared in accordance with the relevant standards described above. The strength tests mentioned above are the subject of a separate publication [1], while the present work focuses on the study and mathematical modeling of the long-term properties of LWAC under cyclic loading. Further details are provided in paper [1].

### 2.3. Types of Testing Machines

**Figure 1:**
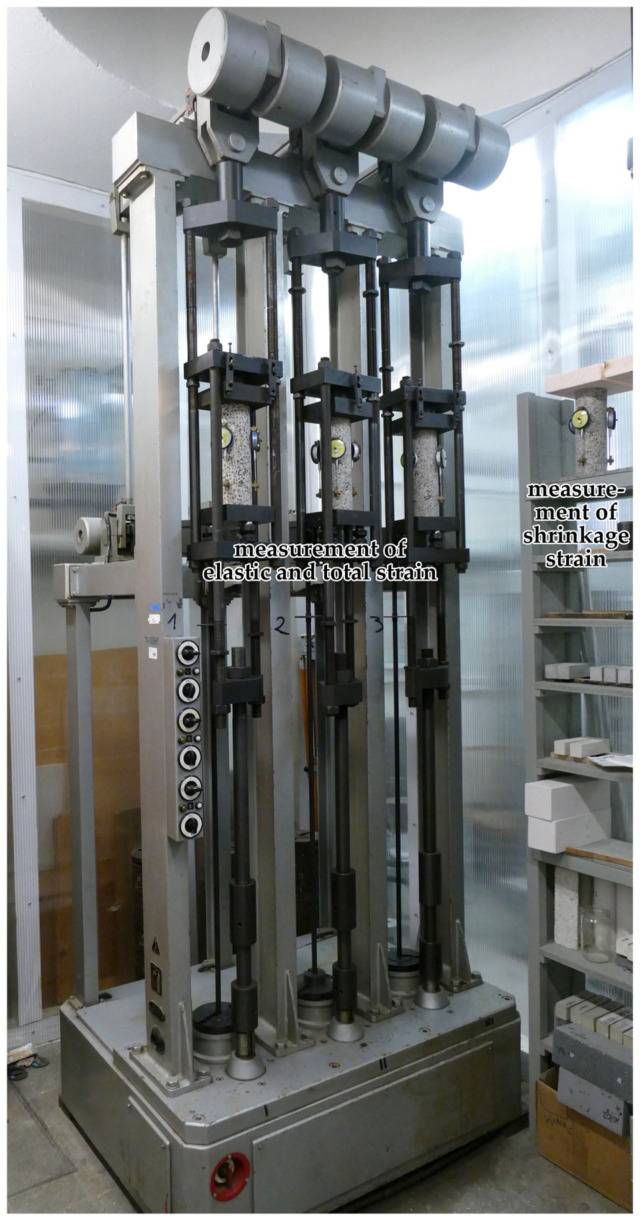
The creep-testing machine (see paper [1]). Testing elastic, creep and shrinkage strains.

The main equipment used to test the long-term mechanical properties of the LWAC under consideration is presented in Figure 1. This figure shows equipment for testing concrete creep and the chamber for testing elastic, creep, and shrinkage strains. The tests were conducted in accordance with the requirements of the relevant standards [84,85,86,87,88,89,90] and Instruction No. 194/88 [86]. According to this instruction, cylindrical samples for testing the axial tensile strength of concrete were glued to steel platens, which were then subjected to tension via ball-and-socket joints. This arrangement allowed for the application of axial force and limited the influence of distortion stresses. The test equipment is of first-class accuracy. Further details are provided in article [1]. Constant environmental conditions were maintained during the long-term tests. Creep and shrinkage tests were conducted in a specialized climatic chamber ensuring stable temperature and relative humidity.

### 2.4. General Schedule for Testing Long-Term Deformation of Concrete Under Cyclic Loading

The creep deformation tests were conducted on six stands in creep-testing machines for the LC1 and LC2 concrete samples. Simultaneously and in the same chamber, shrinkage was tested in accordance with EN 12390-16 [90]. Tests on samples to capture the early-age nonlinear development of concrete properties were conducted at logarithmic time intervals, because at this stage the total strain increases linearly on a logarithmic scale. During the long-term stabilization phase, readings were scheduled in accordance with standard EN 1355 [91]. This enabled the determination of the mechanical parameters of concrete with lightweight sintered aggregate described above [1]. Cubic and cylindrical compressive strengths, as well as axial tensile strength, flexural strength, and splitting strength, were tested at the following intervals: 7, 14, 28, 60, 120, and 300 days. The secant modulus of elasticity tests (see [84]) were performed over a longer period, at the following intervals: 7, 14, 28, 60, 120, 300, 400, 500, and 700 days. Shrinkage and creep strain tests were performed at different intervals. Shrinkage tests using the Amsler method [89] were performed at the following intervals: 1, 3, 7, 14, 21, 28, 56, 90, 120, 150, 190, 270, and 325 days. The shrinkage tests were conducted at intervals consistent with the reference document [89] until the results stabilized. Shrinkage tests in accordance with [90] and creep tests according to Instruction No. 194/88 [86] lasted 1050 days from the collection of cylindrical samples from drill holes in the LC1 concrete block and 1044 days from the collection of samples from the LC2 concrete block. These tests were also conducted at increasingly longer time intervals within each subsequent loading or unloading cycle. For samples taken from the LC1 concrete block, during the first stage of testing, which involved loading for 419 days, shrinkage and creep strain tests were carried out according to the following schedule: day 1: 8 measurements (time *t* = 0 corresponds to the moment of load application; the first reading was taken after 36 min with subsequent readings at one-hour intervals); day 2: 2 measurements (morning and afternoon); from day 3 to 30: 1 measurement per day; from day 33 to 86: 10 measurements every few days; from day 98 to 138: 4 measurements every several days; and from day 160 to 419: 5 measurements every several dozen days. In the second stage of the study, measurements of shrinkage and creep strains were recorded during unloading from days 419 to 572; in the third stage, measurements were recorded during reloading from days 572 to 724; in the fourth stage of the study, measurements were taken during unloading from days 724 to 897; and in the fifth stage, measurements were recorded during the subsequent loading from days 897 to 1050.

**Figure 2:**
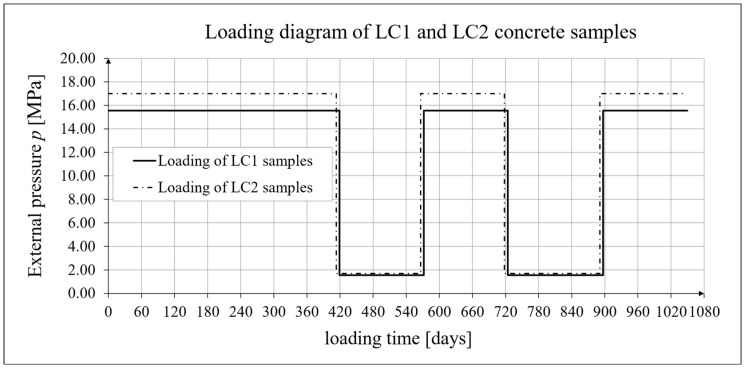
The loading diagram for testing elastic and creep strains.

Shrinkage and creep strain tests on samples taken from the LC2 concrete block were conducted on a very similar schedule (see Figure 2). The drift of the strain sensors was taken into account when determining the expanded measurement uncertainty (see below). In the first stage of the research, strains measurements under loading were carried out for 413 days. In the second stage, measurements of shrinkage and creep strains for this concrete were carried out during unloading from day 413 to day 566. In the third stage, measurements were carried out during reloading from day 566 to day 718. In the fourth stage, measurements were carried out during unloading from day 718 to day 891. In the fifth stage of the research, measurements were carried out during the subsequent loading from day 891 to day 1044.

## 3. Test Results for Long-Term Properties

### 3.1. Test Results of Compressive Strength

**Figure 3:**
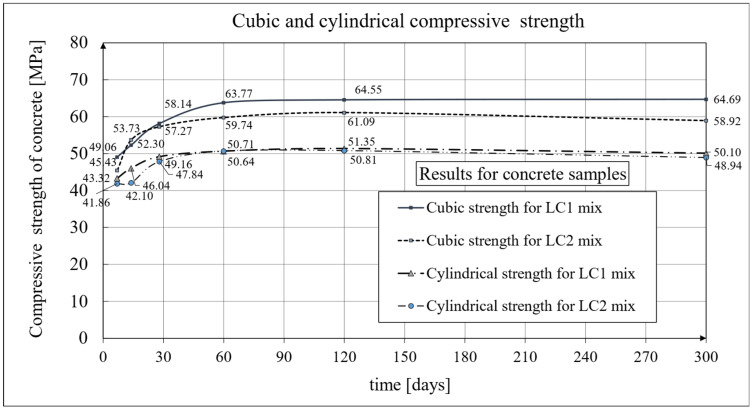
Cubic and cylindrical compressive strength of lightweight concrete according to tests.

The basic strength properties of the concrete with lightweight sintered aggregate (*Certyd*) are described in [1]. The cubic and cylindrical compressive strengths of this lightweight concrete, as determined from the test results, are shown in Figure 3.

The concrete mixes LC1 and LC2, achieved the LC 35/38 class according to EC2:2004 [66] (see Figure 3) seven days after the samples were prepared. At the age of 28 days, the mixes achieved two additional classes, reaching the LC 45/50 class according to EC2:2004 [66] with the D1.8 density class. Concrete cube compressive strength tests were conducted on standard samples with dimensions of 150 × 150 × 150 mm in accordance with standard [85], while cylinder compressive strength tests were conducted on samples with a diameter of *d* = 94 mm and a height of *h* = 190 mm, using samples with the same cross-section as those used for creep testing (and accompanying shrinkage tests). The reported values are averages from tests on three samples, except for the cylinder compressive strength results obtained at 28 days of concrete age, which were averaged from ten samples. These mean strengths were 49.2 MPa for LC1 concrete and 47.8 MPa for LC2 concrete, with standard deviations of 2.49 MPa and 1.21 MPa, respectively. The expanded measurement uncertainty (*U*) of the cube compressive strength did not exceed 1.28% for the LC1 concrete and 1.24% for the LC2 concrete.

### 3.2. Results of Research and Analysis of Secant Modulus of Elasticity

**Figure 4:**
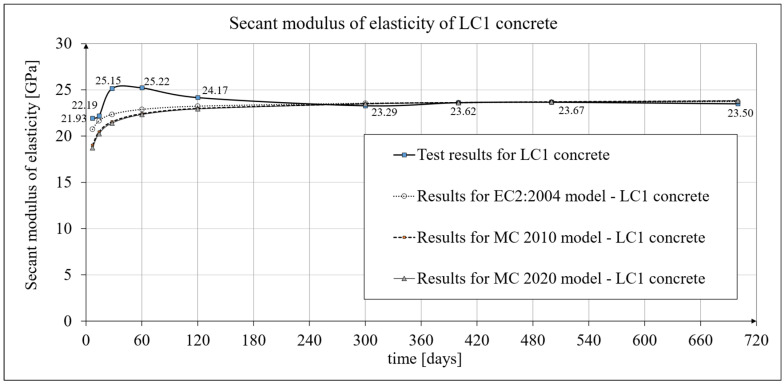
Development of the secant modulus of elasticity for LC1 concrete samples according to the tests and EC2:2004 [66] model, and moreover two models of MC 2010 [15] and MC 2020 [17].

**Figure 5:**
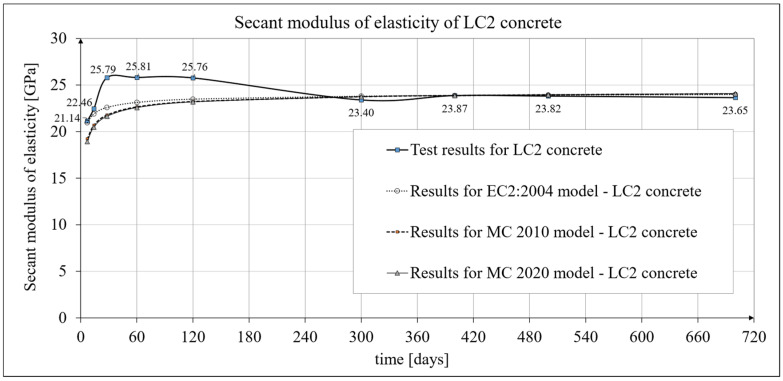
Development of the secant modulus of elasticity for LC2 concrete samples according to the tests and EC2:2004 [66] model, and moreover two models of MC 2010 [15] and MC 2020 [17].

The time-dependent changes in the secant modulus of elasticity of the lightweight sintered aggregate concrete under consideration are described in [1]. The current work focuses primarily on its mathematical models. The results of the secant modulus of elasticity tests are shown in Figure 4 for LC1 concrete and Figure 5 for LC2 concrete. The tests were performed at intervals as specified in Section 2.4. In subsequent tests, the secant modulus of elasticity values stabilized at approximately 23.5 GPa for both LC1 and LC2 concrete. Specimens with the same cross-section as those used for creep tests (and accompanying shrinkage tests) were employed for the secant modulus of elasticity tests. Specimens with a diameter of *d* = 94 mm and a height of *h* = 190 mm were used, and the tests were conducted in accordance with standard [84]. The values presented in Figure 4 and Figure 5 are averages from tests on three samples, except for the secant modulus of elasticity results obtained at 28 days of concrete age, which were averaged from ten samples. These moduli were 25.15 GPa for LC1 concrete and 25.79 GPa for LC2 concrete, with standard deviations of 1.135 GPa and 0.601 GPa, respectively, indicating very good homogeneity in both LC1 and LC2 concrete samples in this regard. The test results for both concrete mixes, LC1 and LC2, showed similar increases over 60 days. In both cases, the secant elastic modulus values increased by approximately 13% between days 7 and 28. The expanded uncertainty of the secant modulus of elasticity measurement, expressed as a percentage, was small and did not exceed 2.03% for LC1 concrete and 1.92% for LC2 concrete.

A comparison of the secant modulus of elasticity development for LC1 concrete samples, based on tests and the EC2:2004 [66] model (see Equation (2)) is shown in Figure 4, while an analogous comparison for LC2 samples is shown in Figure 5 (data used for the EC2:2004 [66] model are provided in Section 4.4). The secant modulus of elasticity development for LC1 concrete samples, based on tests and the MC 2010 [15] and MC 2020 [17] models, is also presented in Figure 4, while the corresponding development for LC2 samples is shown in Figure 5.

The interfaces between the cement matrix and sintered aggregate have a completely different character than those with natural aggregate due to the high water absorption of sintered aggregate. Because curing of the lightweight concrete was completed after 28 days, and because the tests described below showed that shrinkage strains had not yet reached half of their final value, drying shrinkage occurred at the interface between the cement matrix and sintered aggregate, which was significantly greater than at the interface with natural aggregate. This additional shrinkage was so significant that it was not compensated for by an increase in the strength of the cement matrix (as occurs in plain concrete). Therefore, after curing, microcracks may have formed. While they did not significantly affect the compressive strength of the lightweight concrete, they reduced the stiffness of the cement matrix sufficiently to affect the modulus of elasticity of the tested concrete with sintered aggregate (see also Lopez et al. [92]).

### 3.3. Results of Research and Analysis of Shrinkage Strain

**Figure 6:**
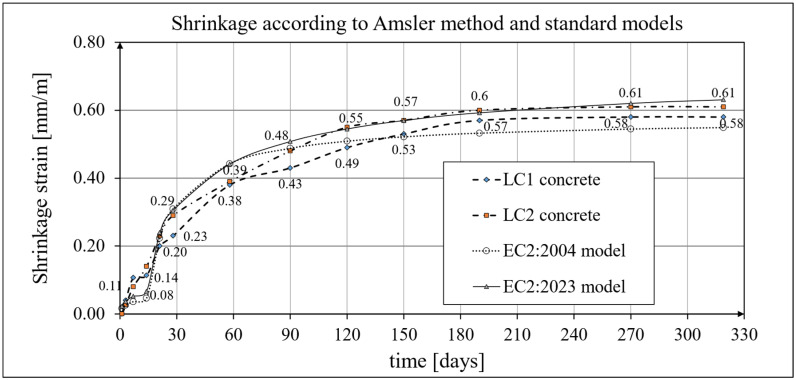
Development of shrinkage according to Amsler method [89], and moreover two models of EC2:2004 standard [66] and EC2:2023 standard [67].

Shrinkage strain measurements for LC1 and LC2 concrete samples were performed using two methods: the first test was performed on three prismatic samples with dimensions of 100 mm × 100 mm × 500 mm using the standard Amsler method [89], while the second test was performed on cylindrical samples with a diameter of *d* = 94 mm and a height of *h* = 3 *d* = 282 mm in accordance with the applicable standard EN 12390-16 [90]. The sample geometry, in accordance with ITB Instruction No. 194/98 [86], was identical to that of the samples used for creep testing. The tests conducted according to the Amsler method [89] were performed at intervals as specified in Section 2.4. The first tests were conducted 24 h after the samples were formed, and the first shrinkage measurements were recorded after 3 days. The experimental results for the development of shrinkage strains in LC1 and LC2 concrete samples, obtained using the Amsler method [89], along with a comparison of the calculated strain development according to the EC2:2004 [66] model (assuming concrete class LC 45/50, *t_s_* = 14 days, and the drying shrinkage factor *η*_3_ = 1.2), are presented in Figure 6.

The shrinkage strain development of samples from both concretes, tested using the Amsler method [89], and the strains calculated according to the EC2:2023 [67] model (assuming a total shrinkage factor *η* = 1.2), are also shown in Figure 6. The LC1 concrete samples showed very low shrinkage between days 7 and 14 of these tests, whereas the LC2 samples showed a constant increase in shrinkage for the first 58 days. Stabilized test results were finally obtained after 270 days for lightweight concrete from both mixes, with the shrinkage of LC1 concrete being slightly lower (see Figure 6).

**Figure 7:**
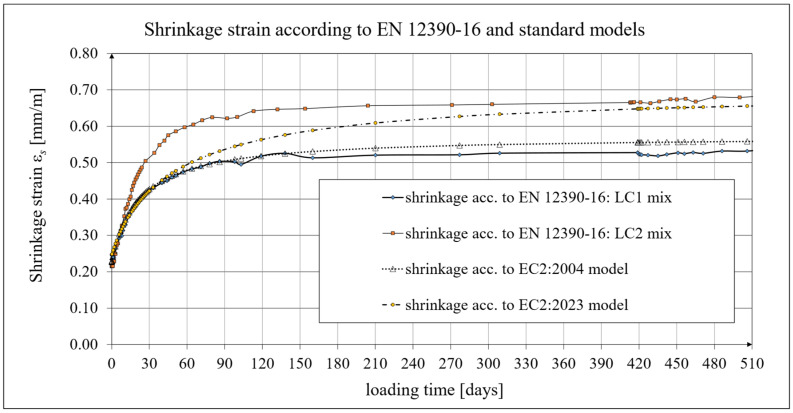
Development of the shrinkage according to standard tests [90], and moreover two models of EC2:2004 standard [66] and EC2:2023 standard [67].

A comparison of concrete shrinkage development from tests according to the EN 12390-16 standard [90] and the EC2:2004 [66] model (assuming concrete class LC 45/50 and *t_s_* = 21 days; see Equation (2)) is shown in Figure 7. The shrinkage tests conducted according to the standard method [90] were performed at intervals as specified in Section 2.4. The shrinkage development according to the standard tests [90] and the MC 2010 [15] model (as well as the MC 2020 [17] and the EC2:2023 [67] model) is also shown in Figure 7. The concrete shrinkage tests conducted according to the current standard [90] lasted 1050 days from sample preparation for concrete from the LC1 mix and 1044 days for concrete from the LC2 mix. The samples were stored in a special chamber in the laboratory where creep and shrinkage measurements were performed simultaneously and where the temperature and humidity were monitored and kept constant (Figure 1). Ultimately, the LC1 concrete shrinkage strain results obtained according to the EN 12390-16 standard [90] were approximately 20% lower than the LC2 concrete shrinkage strain results obtained using the same method. The differences in the *t_s_* values defining the age of concrete at the onset of drying result from different curing conditions of the samples. In tests using the Amsler method, the samples were stored under constant temperature and humidity conditions, but without moistening. The shrinkage strain results obtained are much higher than those reported in [77].

For shrinkage strain tests using the method according to standard [90], the concrete blocks made from both LC1 and LC2 mixtures were stored under analogous conditions; however, the samples were extracted only 28 days after the production of these blocks, which was less favorable for drying of the samples, resulting in a later onset of drying.

### 3.4. Results of Research and Analysis of Creep-Recovery Strain

The strains of LC1 and LC2 concretes were measured as part of the long-term loading program (see Figure 2). Creep measurements on samples from the two concrete mixtures LC1 and LC2 were conducted in both cases on three samples with the following dimensions, in accordance with Instruction 194/98 [86]: diameter *d* = 94 mm and height *h* = 3 *d* = 282 mm (the same type of samples as those used for shrinkage tests using the standard method [90]). The lightweight concrete samples were 28 days old at the time of first loading.

The LC1 concrete samples were initially loaded (on 10 December 2019) with 110 kg weights on the weighing pan, and the LC2 concrete samples (on 16 December 2019) with 120 kg weights—in both cases with a lever ratio of 100. On 9 February 2021, both sample types were unloaded to 10% of the initial force (to 11 and 12 kg, respectively), and measurements of the sample creep deformations continued after this unloading. On 12 July 2021, the samples were reloaded to 100% of the initial force, and on 14 December 2021, both sample types were again unloaded to 10% of the initial force. On 7 June 2022, the samples were loaded again to 100% of the initial force, and the load was maintained until 10 November 2022. Due to the six-day difference in the loading time of the samples (caused by the six-day difference in their preparation times), the creep test under cyclic loading was completed after 1050 days for concrete from the LC1 mix and after 1044 days for concrete from the LC2 mix, using in both cases the three loading intervals and two unloading intervals described above.

**Figure 8:**
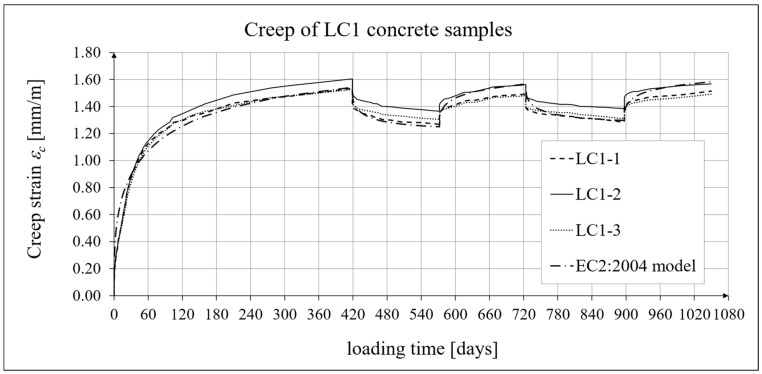
Comparison of creep-recovery strain according to EC2:2004 [66] model and test results of LC1 concrete samples.

**Figure 9:**
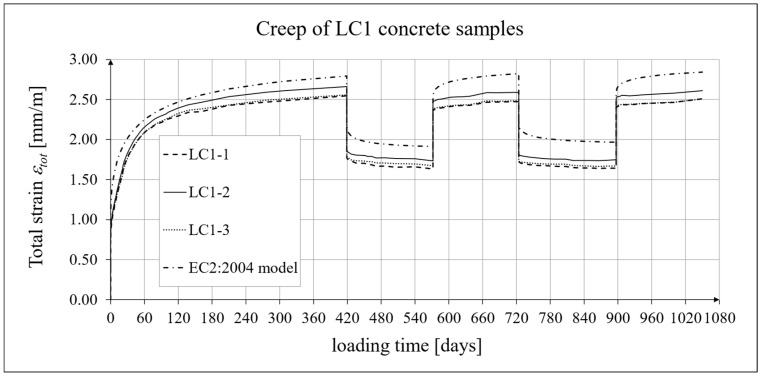
Comparison of total strain according to EC2:2004 [66] model and test results of LC1 concrete samples.

**Figure 10:**
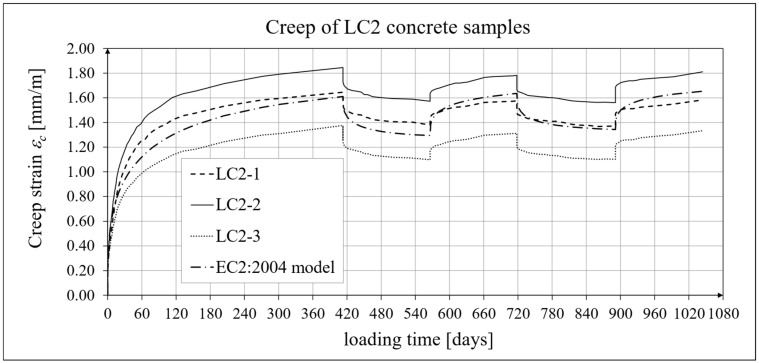
Comparison of creep-recovery strain according to EC2:2004 [66] model and test results of LC2 concrete samples.

**Figure 11:**
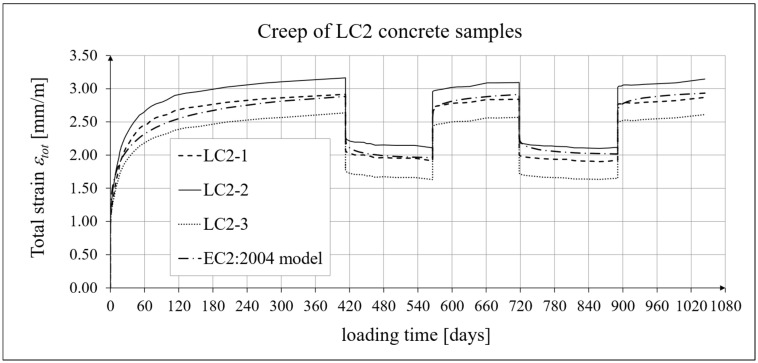
Comparison of total strain according to EC2:2004 [66] model and test results of LC2 concrete samples.

The results of the creep-recovery tests on LC1 concrete samples are shown in Figure 8 and Figure 9, and the results of the creep-recovery tests on LC2 concrete samples are shown in Figure 10 and Figure 11. The following charts show the results of measurements of creep strains only and the total strains of the samples. Creep strains are defined as the difference between total strains and elastic strains plus additional shrinkage strains (measured on cylindrical specimens using the standard method [90] after 28 days of concrete curing). Creep-recovery tests were conducted on three LC1 concrete samples and three LC2 concrete samples, which was due to equipment limitations in the laboratory. For this reason, the strain of each sample was monitored individually, and average values were determined only to calculate the average creep coefficient from the three samples for each of the two concretes.

The creep coefficient, calculated as the ratio of creep strain to elastic strain: *φ* = *ε_c_*/*ε_e_*, was determined in accordance with instructions [86] after a period of not less than one year, although it theoretically refers to time *t* = ∞. The aggregate preparation conditions prior to producing the concrete mix (which occurred outside the ITB laboratory) resulted in the LC2 concrete measurement results exhibiting a certain scatter, attributable to the unintentional inhomogeneity of the LC2 mix.

The measurement uncertainty of total (*ε_tot_*), elastic (*ε_e_*), and creep strains (*ε_c_*), as well as the creep coefficient *φ* = *ε_c_*/*ε_e_* of LC1 lightweight concrete with sintered aggregate after a loading time of *t* = 419 days, is presented in Table 2, where *U* represents the expanded measurement uncertainty, defined as the combined standard measurement uncertainty multiplied by the coverage factor *k* = 2 such that the coverage probability corresponds to approximately 95%. The average value of the creep coefficient (*φ* = *ε_c_*/*ε_e_*) for a loading time of *t* = 419 days was exactly 2.11, with a standard deviation of 0.037.

The measurement uncertainty of total, elastic, shrinkage and creep strains, as well as the creep coefficient of LC2 lightweight concrete with sintered aggregate after the loading time of *t* = 413 days is presented in Table 3, where *U* and *k* are defined as above. The average value of the creep coefficient (*φ* = *ε_c_*/*ε_e_*) for a loading time of *t* = 413 days was exactly 1.94, with a standard deviation of 0.214. The expanded measurement uncertainty (*U*), expressed as a percentage, did not exceed the values presented in Table 4.

Due to the type of sensors used to measure long-term strains, the measurement uncertainties obtained in this case are slightly higher than those obtained when testing strength properties; however, they are still relatively small compared to the scatter of the long-term strain measurement results.

## 4. Models of Long-Term Properties

### 4.1. Secant Modulus of Elasticity Models

The work examines four basic models describing the development of the secant modulus of elasticity of concrete over time: the Eurocode 2 models [66,67] and two models according to pre-standards [15,17]. The value of the creep coefficient (see Section 4.3) according to EC2:2004 [66] is related to the concrete tangent modulus *E_c_*(*t*), which may be taken as a function of the secant modulus of elasticity *E_cm_*(*t*) for concrete as follows:

*E_c_*(*t*) = *α_AC_ E_cm_*(*t*), (1)

where *α_AC_* = *α_EC_*_2_ = 1.05 and the term “EC2:2004 [66] model” refers to the following quantification of the time-dependent secant elastic modulus (as given in EC2 models [66,67]):

*E_cm_*(*t*) = *β_cc_*(*t*)^*n*^ *E_cm_*, (2)

where

*E_cm_*— the secant modulus of elasticity for concrete at the age of 28 days;
*β_cc_*(*t*)— a coefficient that depends on the age of the concrete *t* and represents the evolution of concrete properties over time (see Equation (3.2) in EC2:2004 [66] and Equation (B.2) in EC2:2023 [67]; Equation (B.2) has a slightly more complex form than Formula (3.2) in EC2:2004 [66] and contains an additional parameter: *t_ref_*);
*t*— the age of the concrete in days;
*t_ref_*— a reference time for assessing the compressive strength of concrete, generally other than 28 days;
*s*— a coefficient (see Equation (3.2) in EC2:2004 [66]) that depends on the type of cement (*s* = 0.25 for the considered cement of strength Class CEM 42.5 N (Class N));
*s_c_*— a coefficient (see Equation (B.2) in EC2:2023 [67]) that depends on the concrete strength and its early development, i.e., on the type of cement (*s_c_* = 0.4 for a concrete strength of 35 MPa < *f_ck_* < 60 MPa and the applied cement class CEM 42.5 N (Class CN)), where the considered lightweight concrete class LC 45/50 corresponds to a compressive strength *f_lck_* = 45 MPa;
*n*— the power exponent in Expression (2); *n* = 0.3 is assumed in the case of the standard model according to EC2:2004 [66] and *n* = 1/3 according to EC2:2023 [67].

In the case of the model according to the MC 2010 pre-standard [15], instead of the *E_c_*(*t*) modulus, the *E_ci_*(*t*) modulus is used, which represents the mean value of the tangential modulus of elasticity of concrete. The relationship between this modulus and the secant modulus of elasticity *E_cm_*(*t*) can be described by a formula analogous to Formula (1), assuming a coefficient *α_AC_* = *α_MC_* with a variable value depending on the concrete class. However, for the LC45 class (*f_lck_* = 45 MPa) according to the MC 2010 pre-standard [15], a value of *α_AC_* = *α_MC_* = 1.086 can be assumed, and *n* = 0.5.

For the most recent model, according to the MC 2020 pre-standard [17], it was assumed that *n* = 1/3 and the coefficient *α_AC_* = *α_EN_* = 1.05 (MC 2020 [17]). Furthermore, the formula determining the coefficient *β_cc_*(*t*) takes the form of Equation (B.2) in EC2:2023 [67] and contains an additional parameter *t_ref_* as explained above. However, in the present tests, the time used to assess the compressive strength of concrete is 28 days, so the coefficient *β_cc_*(*t*) can also be determined in this case using Formula (3.2) in EC2:2004 [66], although with *s_c_* = 0.4. The development of the secant modulus of elasticity for the LC1 and LC2 concrete mixtures according to the tests and the EC2:2004 [66] model is shown in Figure 4 and Figure 5, respectively. The development of the secant modulus of elasticity for concrete mixes LC1 and LC2 based on the tests and the two models MC 2010 [15] and MC 2020 [17] (identical to the EC2:2023 [67] model) is also presented in Figure 4 and Figure 5, respectively.

### 4.2. Shrinkage Models

Concrete shrinkage has been the subject of many studies; one of the earliest models was the Glanville model [93,94]. Subsequently, more complex models were developed—these are the models included in standards and pre-standards. The term “EC2:2004 [66] model” refers to the concrete shrinkage model described in the EC2:2004 [66] standard, as described below. The value of the total shrinkage strain *ε_cs_*(*t*) is determined using the formula:

*ε_cs_*(*t*) = *ε_cd_*(*t*) + *ε_ca_*(*t*), (3)

where:

*ε_cs_*(*t*)— the total shrinkage strain;
*ε_cd_*(*t*)— the shrinkage strain due to drying;
*ε_ca_*(*t*)— the autogenous shrinkage strain.

*ε_cd_*(*t*) = *β_ds_*(*t*, *t_s_*) *k_h_* *ε*_*cd*,0_, (4)

where:

*β_ds_*(*t*, *t_s_*)— the function describing the development of shrinkage strains due to drying as given by Equation (3.10) given in EC2:2004 [66];
*t*— the age of the concrete at the moment in question, in days;
*t_s_*— the age of the concrete (in days) at the beginning of drying (or swelling); this is usually at the end of curing;
*h*_0_— the nominal cross-sectional dimension [mm]; *h*_0_ = 2 *A*_c_/*u* (see Equation (3.10) in EC2:2004 [66]);
*A_c_*— the concrete cross-sectional area;
*u*— the perimeter of the part of the cross-section exposed to drying;
*k_h_*— a coefficient depending on the nominal size *h*_0_ in accordance with Table 3.3 in EC2:2004 [66].

The nominal unrestrained shrinkage strain due to drying *ε_cd_*_,0_ is determined by Formula (B.11) given in EC2:2004 [66].

The development of autogenous shrinkage strain is determined by the formula:

*ε_ca_* (*t*) = *β_as_*(*t*) *ε_ca_* (∞), (5)

where the final value of the autogenous shrinkage strain *ε_ca_* (∞) is described by Equation (3.12) in EC2:2004 [66], and the function characterizing the development of autogenous shrinkage *β_as_*(*t*) is described by Equation (3.13), also in EC2:2004 [66].

For the models according to the MC 2010 pre-standard [15] and the MC 2020 pre-standard [17], certain generalizations were introduced compared to the model according to EC2:2004 [66]. The principle of superposition of shrinkage strains was maintained, but instead of the concept of autogenous shrinkage strain, the concept of basic shrinkage was introduced. Basic shrinkage was defined similarly to *ε_ca_*(*t*) in Equation (5), except that instead of *ε_ca_*(∞), the basic notional shrinkage coefficient *ε_cas_*_0_(*f_cm_*) was introduced, which is defined by the formula:

(6) $$\varepsilon_{cas0}(f_{cm})=-\alpha_{as}{\left(\frac{f_{cm}/10}{6\;+\;f_{cm}/10}\right)}^{2.5}\cdot 10^{-6}$$

The development of shrinkage strain due to drying is determined by the formula:

*ε_cds_*(*t*, *t_s_*) = *ε*_*cds*0_(*f_cm_*) *β_RH_*(*RH*) *β_ds_*(*t* − *t_s_*), (7)

where the individual components of the product are determined in a modified manner relative to Formula (7) contained in EC2:2004 [66]. The notional drying shrinkage coefficient *ε_cds_*_0_(*f_cm_*)

(8) $$\varepsilon_{cds0}(f_{cm})=\left[\left(220\;+110\alpha_{ds1}\right)\cdot \exp\left(-\alpha_{ds2}f_{cm}\right)\right]\cdot 10^{-6},$$

(9a) $$\beta_{RH}=-1.55\;\left[1-{\left(\frac{RH}{RH_{0}}\right)}^{3}\right]\;\mathrm{for}\;40\%\leq RH<99\%\cdot \beta_{s1},$$

or

*β_RH_* = 0.25 for *RH* ≥ 99% · *β_s_*_1_, (9b)

(10) $$\beta_{ds}(t-t_{s})={\left(\frac{t-t_{s}}{t-t_{s}+\;0.035{h_{0}}^{2}}\right)}^{0.5},$$

(11) $$\beta_{s1}={\left(\frac{35}{f_{cm}}\right)}^{0.1}\leq 1.0,$$

*α_ds_*_1_—a coefficient depending on the type of cement (see EC2:2004 Section 3.1.2(6) [66]);
For the concrete under consideration *α_ds_*_1_ = 4.0 was assumed for the cement class N;
*α_ds_*_2_—a coefficient depending on the type of cement;
For the concrete under consideration *α_ds_*_2_ = 0.012 was assumed for the cement class N;
*RH*—the ambient relative humidity [%], *RH*_0_ = 100%.

For the model according to the MC 2020 pre-standard [17] and the cement class CEM I 42.5 N used in the tests, the differences compared to the model according to the MC 2010 pre-standard [15] are small and largely concern only the notation. In the latter model, the shrinkage values are negative (although this is also a matter of convention). The values of the nominal drying shrinkage strain are determined by Formula (B.21) in MC 2020 [17], which is equivalent to Formula (8), in which the value *α_ds_*_2_ = 0.012 is directly substituted, and the values of the function *β_ds_* (*t* − *t_s_*) are determined by Formula (B.24) in MC 2020 [17], which is equivalent to Formula (10). The most significant difference concerns the humidity coefficient *β_RH_*, which is determined from modified formulas that contain the additional coefficients *a* and *b*. However, the MC 2020 pre-standard [17] does not provide the coefficient values. The method for determining the *β_RH_* coefficient (see Formula (9a)) can be used based on the equivalent Formula (5.1–81) given in MC 2010 [15], remembering the sign in this formula. Differences in the approaches according to both pre-standards are evident for high humidity, but this is not the case considered in this study.

Considering the pre-standard models according to MC 2010 [15] and MC 2020 [17], the values of the total shrinkage strain *ε_cs_*(*t*, *t_s_*), which are determined from a formula analogous to Equation (3), should be multiplied in the case of lightweight concrete (in accordance with Formula (5.1–84) of MC 2010 [15] and in accordance with Table M2 of MC 2020 [17]) by the coefficient *η*, which for lightweight concrete of class LC20 and higher (as is the case for the classes of lightweight concrete considered herein) is *η* = 1.2.

### 4.3. Creep-Recovery Models—Parameterization and Modeling of Constitutive Relationships

- Integral equations of the hereditary law

The analysis of creep strains was carried out based on several long-term models, while the presented graphs show the results obtained using the creep models described below according to EC2:2004 [66] and the two pre-standards: MC 2010 [15] and MC 2020 [17]. The EC2:2004 [66] standard model has the traditional form of a product of functions, the first of which represents the basic creep coefficient (its final value), while the remaining functions account for factors considered to significantly affect the development of concrete strain over time, as discussed in detail in the monograph by A. M. Neville [26]. In the MC 2010 pre-standard [15], modified formulas for calculating the concrete creep coefficient were presented. These formulas express the total creep coefficient as the sum of two components: basic creep and drying creep (see below). These modified formulas were retained in the MC 2020 pre-standard [17] and the EC2:2023 standard [67] model. The methods described here for determining long-term constitutive relationships and their parameters governing the development of strains in lightweight structural concrete with sintered aggregate have been developed. Through research on the phenomenon of partially reversible creep strains in structural concrete with lightweight sintered aggregate, it was possible to develop a number of procedures for analyzing test results and classifying the actual long-term characteristics of concrete made with lightweight mixes according to appropriate long-term models, such as:

- The hereditary model with concrete aging (and its changing modulus of elasticity);
- The elastic hereditary model;
- The concrete aging model;
- Standard and pre-standards models.

According to Boltzmann’s principle of superposition [95], the total strain ε(t) is a superposition of strain increments $\Delta \varepsilon_{i}(t,\tau )$ , where *τ* denotes the times at which stress increments are applied. It is assumed that from the time *t* = 0, the stress increments $\Delta \sigma_{i}(\tau )$ are gradually applied at times *t* = *t*_0_, *t*_1_, *t*_2_, …, corresponding to the individual strain increments $\Delta \varepsilon_{i}(t,\tau )$ , where the proportionality of the mutual increments of both quantities is reflected by the creep function (memory function) denoted by the symbol J(t,τ) . Thus, the expression takes the form:

(12) $$\varepsilon (t)=\sum_{i=0}^{n}\Delta \varepsilon_{i}\left(t,\tau \right)=\sum_{i=0}^{n}\Delta \sigma_{i}\left(t,\tau \right)\;J\left(t,\tau \right)$$

where the creep function (the compliance function) has the form (see [95]):

(13) $$J(t,\tau )=\frac{1}{E(\tau )}+C(t,\tau )$$

Here, *E*(*τ*) is the time-varying modulus of elasticity of concrete (see Section 4.1) and *C*(*t*,*τ*) is a creep measure defined below when discussing the individual creep models. If the stress increments Δ*σ_i_*(*τ*) occur at closely spaced time intervals, then they can be described as a continuous function of time, and the superposition principle can be presented in the form of the integral equation of the hereditary law of Volterra [95] for a uniaxial state:

(14) $$\varepsilon (t)=\int_{t_{0}}^{t}\frac{\partial \sigma (\tau )}{\partial \tau }\;J(t,\tau )\;d\tau$$

In many publications and standard documents such as MC 2010 [15] or MC 2020 [17], the classical Volterra integral equation was modified by adding to the right side of the equation a term expressing the growth of strain of the body at time *t*, occurring after applying a stress that remains constant over time *σ*(*t*_0_) = const at time *t*_0_, yielding the form:

(15) $$\varepsilon (t)=\sigma (t_{0})\;J(t,t_{0})+\int_{t_{0}}^{t}\frac{\partial \sigma (\tau )}{\partial \tau }\;J(t,\tau )\;d\tau .$$

Formulations of the compliance function *J*(*t*,*τ*) in the form of Equation (13) with concrete aging and its changing modulus of elasticity can be specified for the individual long-term models according to Eurocode 2, and the two above-mentioned pre-standards and are presented below as part of the descriptions of the individual models.

Thus, Expression (14) takes the form:

(16) $$\varepsilon (t)=\int_{t_{0}}^{t}\frac{\partial \sigma (\tau )}{\partial \tau }\;\left[\frac{1}{E(\tau )}+C(t,\tau )\right]\;d\tau ,$$

while Expression (15) takes the form:

(17) $$\varepsilon (t)=\sigma (t_{0})\;\left[\frac{1}{E(t_{0})}+C(t,t_{0})\right]+\int_{t_{0}}^{t}\frac{\partial \sigma (\tau )}{\partial \tau }\;\left[\frac{1}{E(\tau )}+C(t,\tau )\right]\;d\tau .$$

Let us assume that at the concrete setting age *t*_0_, a constant stress *σ*(*t*_0_) is applied, which acts until time *t*_1_. Then the strains at interval from *t*_0_ to *t*_1_ can be described as follows:

(18) $$\varepsilon (t)=\sigma (t_{0})\left[\frac{1}{E(t_{0})}+C(t,t_{0})\right],$$

Suppose that at time *t*_0_, a stress *σ*(*t*_0_) is applied, which acts until time *t*_1_, when a stress *σ*(*t*_1_) is applied. The stress variability at the time interval from *t* = *t*_0_ to *t* = *t*_2_ > *t*_1_ can be described using the Heaviside function (cf. [95,96]):

(19) $$\sigma (t)=\sigma (t_{0})\;\left[1-H(t-t_{1})\right]+\sigma (t_{1})\;\left[H(t-t_{1})\right].$$

The partial derivative of the function *σ*(*τ*) with respect to *τ* takes the form:

(20) $$\frac{\partial \sigma (\tau )}{\partial \tau }=\sigma (t_{0})\;\left[-\delta (\tau -t_{1})\right]+\sigma (t_{1})\;\left[\delta (\tau -t_{1})\right]=\left(\sigma (t_{1})\;-\sigma (t_{0})\;\right)\;\delta (\tau -t_{1}).$$

Hence, after substituting (20) into (17), for creep-recovery strains at the interval from *t*_1_ to *t*_2_, we obtain the following:

(21) $$\varepsilon (t)=\sigma (t_{0})\left[\frac{1}{E(t_{0})}+C(t,t_{0})\right]+\left(\sigma (t_{1})-\sigma (t_{0})\right)\left[\frac{1}{E(t_{1})}+C(t,t_{1})\right].$$

Let us assume, in turn, that at time *t*_0_, a stress *σ*(*t*_0_) is applied, which acts until time *t*_1_, when a stress *σ*(*t*_1_) is applied, which, in turn, acts until time *t*_2_, when a stress *σ*(*t*_2_) is applied. The change in stress over time can also be described using the Heaviside function:

(22) $$\sigma (t)=\sigma (t_{0})\;\left[1-H(t-t_{1})\right]+\sigma (t_{1})\left[H(t-t_{1})-H(t-t_{2})\right]+\sigma (t_{2})\;\left[H(t-t_{2})\right].$$

The partial derivative of the function *σ*(*τ*) with respect to *τ* now has the form:

(23) $$\frac{\partial \sigma (\tau )}{\partial \tau }=\left(\sigma (t_{1})\;-\sigma (t_{0})\;\right)\;\delta (\tau -t_{1})+\left(\sigma (t_{2})\;-\sigma (t_{1})\;\right)\;\delta (\tau -t_{2}).$$

Hence, after substituting (23) into (17), for creep-recovery strains at the interval from *t*_2_ to *t*_3_, we obtain the following equation:

(24) $$\varepsilon (t)=\sigma (t_{0})\left[\frac{1}{E(t_{0})}+C(t,t_{0})\right]+\left(\sigma (t_{1})-\sigma (t_{0})\right)\left[\frac{1}{E(t_{1})}+C(t,t_{1})\right]+\left(\sigma (t_{2})\;-\sigma (t_{1})\right)\left[\frac{1}{E(t_{2})}+C(t,t_{2})\right].$$

Therefore, proceeding recursively, for creep-recovery strains at the interval from *t_n_* to *t_n_*_+1_, we obtain the following equation:

(25) $$\varepsilon (t)=\sigma (t_{0})\left[\frac{1}{E(t_{0})}+C(t,t_{0})\right]+\sum_{i=1}^{n}\left(\sigma (t_{i})-\sigma (t_{i-1})\right)\left[\frac{1}{E(t_{i})}+C(t,t_{i})\right]$$

A mathematically similar formulation was obtained by Bažant and Jirásek (see [97,98]), defined as the Age Adjusted Effective Modulus (AAEM). Let us consider again the description of stress variation over time, for example, in the form (22). The necessity of using the integral equation in the form (15), i.e., the need to distinguish an initial term, resulted from the lack of an initial condition (for time *t* = *t*_0_) in Equations (19) and (22). By applying such a condition using the Heaviside function, the description of stress variability can be presented in the form:

(26) $$\sigma (t)=\sigma (t_{0})\;\left[H(t-t_{0})-H(t-t_{1})\right]+\sigma (t_{1})\left[H(t-t_{1})-H(t-t_{2})\right]+\sigma (t_{2})\;\left[H(t-t_{2})\right].$$

The partial derivative of the function *σ*(*τ*) with respect to *τ* now takes the form:

(27) $$\frac{\partial \sigma (\tau )}{\partial \tau }=\sigma (t_{0})\;\delta (\tau -t_{0})+\left(\sigma (t_{1})\;-\sigma (t_{0})\;\right)\;\delta (\tau -t_{1})+\left(\sigma (t_{2})\;-\sigma (t_{1})\right)\;\delta (\tau -t_{2}).$$

Hence, after substituting (27) into (16), for strains at the interval from *t*_2_ to *t*_3_, we obtain the (correct) Equation (24) again. This means that correcting the Boltzmann–Volterra superposition principle (provided that the initial condition is correctly formulated) is unnecessary; this correction is nonetheless cited in many documents, including MC 2010 [15] and MC 2020 [17]. Therefore, the integral equations in the form (15) or (17) are redundant.

In the present study, three constitutive models were considered for comparative purposes.

- Generalized EC2:2004 [66] model—the concrete creep model described in EC2:2004 [66], in which the creep measures can be determined

The creep coefficient *φ*(*t*, *t*_0_) can be calculated from the formula:

*φ*(*t*, *t*_0_) = *φ*_0_ · *β_c_*(*t*, *t*_0_). (28)

wherein

*φ*_0_— the notional creep coefficient, which may be estimated from:
  *φ*_0_ = *φ_RH_* · *β*(*f_cm_*) · *β*(*t*_0_). (29)

In the above formula:

*φ_RH_*— the coefficient accounting for the influence of relative humidity in accordance with the Equation (B.3) in EC2:2004 [66];
*β*(*f_cm_*)— the coefficient accounting for the influence of the mean compressive concrete strength at 28 days in accordance with the Equation (B.4) in EC2:2004 [66];
*β*(*t*_0_)— a factor to allow for the effect of concrete age at loading on the notional creep coefficient in accordance with the Equation (B.4) in EC2:2004 [66];
*h*_0_— the nominal cross-sectional dimension in [mm] as in Section 4.2 (see Equations (B.3a/b) and (B.8a/b) in EC2:2004 [66]);
*β_c_* (*t*, *t*_0_)— a coefficient describing the development of creep with time after loading, which may be estimated using Expression (B.7) in EC2:2004 [66];
*β_H_*— a coefficient (see Equation (B.7) in EC2:2004 [66]) depending on the relative humidity (*RH* in %) and the notional member size (*h*_0_), which may be estimated from the Equations (B.8a/b) in EC2:2004 [66], in which in turn
*α*_1_, *α*_2_, and *α*_3_— are coefficients (see Equations (B.3b) and (B.8b) in EC2:2004 [66]) that consider the influence of the concrete strength (*f_cm_*).

The value of *φ*(*t*, *t*_0_) given above is related to the tangent modulus of elasticity *E_c_*(*t*_0_), which may be taken as given by Equation (1). The method of determining the value of the secant elastic modulus *E_cm_*(*t*) is given in Section 4.1, and on this basis, the creep strains of concrete are determined. The creep strain of concrete *ε_cc_*(∞, *t*_0_) at time *t* = ∞ for a constant compressive stress *σ_c_* applied at the concrete age *t*_0_ is given by:

*ε_cc_*(∞, *t*_0_) = *φ*(∞, *t*_0_) · (*σ_c_*/*E_c_*(*t*_0_)). (30)

The generalization of the standard model according to EC2:2004 [66] consisted of formulating the creep measures in such a way that the characteristics of the creep coefficient (28) were transferred from the interval from *t*_0_ to *t*_1_ to the interval from *t*_1_ to *t*_2_ and beyond. At the interval from *t*_0_ to *t*_1_, the creep measure is determined according to the formula (see MC 2020 [17]):

*C*_0_(*t*, *t*_0_) = *φ*(*t*, *t*_0_)/*E_c_*_0_(*t*_0_). (31)

where *E_c_*_0_(*t*_0_) is the modulus of elasticity at the time of loading *t*_0_ and the strains at the interval from *t*_0_ to *t*_1_ are obtained according to Formula (18).

By the same principle, at the interval from *t_i_* to *t*_*i* + 1_:

*C_i_*(*t*, *t_i_*) = *φ*(*t*, *t_i_*)/*E_ci_*(*t_i_*) (32)

and the strains at the interval from *t_n_* to *t*_*n* + 1_ are obtained according to Formula (25).

The influence of the type of cement on the creep coefficient of concrete can be accounted for by modifying the age of the concrete at the moment of loading *t*_0_ = *t*_0,*adj*_ according to Expression (B.9) in EC2:2004 [66], but for class N cements, the exponent *α* in this expression depends on the type of cement: *α* = 0.

The impact of increased or decreased temperature within the range of 0–80 °C on the maturity of concrete can be accounted for by the so-called adjusted concrete age *t*_0,*T*_, i.e., adjusted to the temperature in accordance with Expression (B.10) of the EC2:2004 [66] standard. The tests were conducted at room temperature, and modification of the concrete age at the moment of loading in accordance with Expressions (B.9) and (B.10) according to EC2:2004 [66] was not necessary.

- Generalized MC 2010 [15] model—the concrete creep model described in the MC 2010 pre-standard [15], in which the creep measures can be determined

In the models discussed below according to the MC 2010 pre-standard [15] and the MC 2020 pre-standard [17], similar assumptions were made, which constitute a certain generalization of the creep model according to EC2:2004 [66], consisting mainly of the separating the total creep coefficient into two components, i.e., the basic creep coefficient *φ_bc_*(*t*, *t*_0_) and the drying creep coefficient *φ_dc_*(*t*, *t*_0_), which in turn are determined separately (see [15,17]), and the result was presented as a sum of two functions (not a product). The new formula for creep of concrete in MC 2010 [15] was described in detail by Hołowaty [99]. For the model according to the MC 2010 pre-standard [15], it is assumed that the creep strain of concrete *ε_cc_* (*t*, *t*_0_) for a constant compressive stress *σ_c_*(*t*_0_) applied at time *t*_0_, is defined by the equation:

*ε_cc_*(*t*, *t*_0_) = *φ*(*t*, *t*_0_) · (*σ_c_*(*t*_0_)/*E_ci_*), (33)

where *E_ci_* is taken as the mean value of the tangent modulus of elasticity for concrete at the age of 28 days; hence, *E_ci_* = *E_cm_*, and in addition:

*φ*(*t*, *t*_0_) = *φ_bc_*(*t*, *t*_0_) *+ φ_dc_*(*t*, *t*_0_), (34)

*φ_bc_*(*t*, *t*_0_) = *β_bc_*(*f_cm_*) *β_bc_*(*t*, *t*_0_), (35)

*φ_dc_*(*t*, *t*_0_) = *β_dc_*(*f_cm_*) *β*(*RH*) *β_dc_*(*t*_0_) *β_dc_*(*t*, *t*_0_), (36)

where

(37) $$\beta_{bc}\left(f_{cm}\right)=\frac{1.8}{{\left(f_{cm}\right)}^{\;0.7}},$$

(38) $$\beta_{bc}(t,t_{0})=\ln\left[\;{\left(\frac{30}{t_{0,adj}}+0.035\right)}^{2}\left(t-t_{0}\right)+1\right],$$

where *t*_0,*adj*_ denotes the adjusted concrete age—just like the value *t*_0_ in Formula (B.9) in EC2:2004 [66],

(39) $$\beta_{dc}\left(f_{cm}\right)=\frac{412}{{\left(f_{cm}\right)}^{\;1.4}},$$

(40) $$\beta \left(RH\right)=\frac{1-\frac{RH}{RH_{0}}}{0.1\;\sqrt[{3}]{h_{0}}},$$

in which the value *RH*_0_ = 100 [%] was immediately assumed, and *h*_0_ is the notional dimension of the element in mm, determined by the formula *h*_0_ = 2 *A*_c_/*u* (see above), and according to the pre-standards MC 2010 [15] and MC 2020 [17], two different designations for this quantity were adopted, i.e., *h* in MC 2010 [15] and *h_n_* in MC 2020 [17].

(41) $$\beta_{dc}(t_{0})=\frac{1}{0.1+{t_{0,adj}}^{0.2}},$$

(42) $$\beta_{dc}(t,t_{0})={\left(\frac{\left(t-t_{0}\right)}{\beta_{h}+\left(t-t_{0}\right)}\right)}^{\gamma \left(t_{0}\right)},$$

where, in turn,

(43) $$\gamma (t_{0})=\frac{1}{2.3+\frac{3.5}{\sqrt{t_{0,adj}}}},$$

*β_h_* = 1.5*h*_0_ + 250*α _fcm_* ≤ 1500*α _fcm_*, (44)

(45) $$\alpha_{f_{cm}}={\left(\frac{35}{f_{cm}}\right)}^{0.5}.$$

The influence of the type of cement on the creep coefficient of concrete can be accounted for by modifying the age of the concrete at the moment of loading *t*_0_, replacing it with the time *t*_0,*adj*_ according to the following expression, which here takes a slightly different form than Formula (5.1–73) in MC 2010 [15]:

(46) $$t_{0,adj}=t_{0,T}{\left[\;\frac{9}{2+{t_{0,T}}^{1.2}}+1\right]}^{\alpha }\;\geq \;0.5\mathrm{days},$$

*t*_0,*T*_ is the age of the concrete at the moment of loading adjusted to the temperature [in days], depending on the temperature history during maturation; the formula for *t*_0,*T*_ complies with Formula (5.1–85) of the MC 2010 pre-standard [15] and corresponds to Expression (B.10) of the EC2:2004 standard [66]. At room temperature (as in the experiments performed), *t*_0,*T*_ = *t*_0_ is assumed. Taking into account the type of cement used in the tests, i.e., cement with strength class CEM 42.5 (class N), the power exponent *α* in Expression (46) was assumed as: *α* = 0. At the interval from *t*_0_ to *t*_1_, the creep measure is determined according to Formula (31), and the strains at this interval are obtained according to Formula (18). In the same way, the creep measure at the interval from *t_i_*_−1_ to *t_i_* is determined according to Formula (32), and the strains at this interval are obtained according to Formula (25).

- Generalized MC 2020 [17] model—the concrete creep model described in the MC 2020 pre-standard [17], in which the creep measures can be determined

The model according to the pre-standard MC 2020 [17] does not differ substantially from the model according to the EC2:2023 standard [67] in terms of determining creep strains, so both models have been treated as equivalent here.

For the model according to the pre-standard MC 2020 [17] and the cement class CEM I 42.5 N used in these tests, the differences from the model according to the pre-standard MC 2010 [15] are small and largely concern only the notation. For the model according to the MC 2020 pre-standard [17], similar assumptions were made regarding the superposition of creep strains as for the MC 2010 pre-standard [15]:

*φ* (*t*, *t*_0_) = *φ_bc_*(*t*, *t*_0_) *+ φ_dc_*(*t*, *t*_0_), (47)

*φ_bc_*(*t*, *t*_0_) = *β_bc_*(*f_cm_*) *β_bc_*(*t*, *t*_0_), (48)

(in the original text (MC 2020): *φ_bc_*(*t*, *t*_0_) = *β_bc_*_,*fcm*_ · *β_bc_*_,*t−t*0_),

*φ_dc_*(*t*, *t*_0_) = *β_dc_*(*f_cm_*) *β* (*RH*) *β_dc_*(*t*_0_) *β_dc_*(*t*, *t*_0_), (49)

(in the original text (MC 2020): *φ_dc_*(*t*, *t*_0_) = *β_dc_*_,*fcm*_
*· β_dc_*_,*RH*_
*· β_dc_*_,*t*0_
*· β_dc_*_,*t−t*0_).

The above quantities: *β_bc_*(*f_cm_*) = *β_bc_*_,*fcm*_, *β_bc_*(*t*, *t*_0_) = *β_bc_*_,*t−t*0_, *β_dc_*(*f_cm_*) *= β_dc_*_,*fcm*_, *β* (*RH*) = *β_dc_*_,*RH*_, *β_dc_*(*t*_0_) = *β_dc_*_,*t*0_, and *β_dc_*(*t*, *t*_0_) = *β_dc_*_,*t−t*0_ can be determined using Formulas (37)–(42), respectively, where *γ*(*t*_0,_ *_adj_*), *β_h_*, and *α_fcm_* are defined by Formulas (43)–(45), respectively; as for MC 2010 [15].

The influence of the type of cement on the creep coefficient of concrete can be accounted for as above by modifying the age of the concrete at the moment of loading *t*_0_, replacing it with the time *t*_0,*adj*_ according to Expression (46), in which *t*_0,*T*_ is the age of the concrete at the moment of loading adjusted to the temperature history during curing, which is determined by a formula that corresponds to Expression (B.10) of the EC2:2004 standard [66] and is consistent with Formula (5.1–85) of the MC 2010 pre-standard [15], Formula (14.11–1) of the MC 2020 pre-standard [17] and Formula (B.18) of the EC2:2023 standard [67].

The difference between Formula (46) and Formula (B.17) of the EC2:2023 standard [67] is only that the symbol *α* has been replaced by the symbol *α_SC_*, which, however, takes the same values according to the pre-standard MC 2010 [15]. Taking into account the type of cement used in the tests, i.e., cement with strength class CEM 42.5 (class N), the power exponent *α* in Expression (46) was assumed: *α* = 0 (*α_SC_* = 0), except that at room temperature (as in the tests performed), *t*_0,*T*_ = *t*_0_ is assumed, therefore *t*_0,*adj*_ = *t*_0_. In the interval from *t*_0_ to *t*_1_, the creep measure is determined according to Formula (31), and the strains in this interval are obtained according to Formula (18). In the same way, the creep measure at the interval from *t_i_*_−1_ to *t_i_* is determined according to Formula (32), and the strains in this interval are obtained according to Formula (25).

When using the model according to EC2:2004 [66], the creep coefficient values for lightweight aggregate concrete should be reduced by a correction factor expressed as (*ρ*/2200)^2^, where *ρ* [kg/m^3^] is the density of the oven-dried concrete. For the lightweight concretes under consideration, LC1 and LC2, with density class D1.8 (1800 kg/m^3^), this yields a factor with an approximate value of 0.67. The same value of the reduction factor *η_E_* = 0.67 is obtained for the models according to MC 2010 [15] and MC 2020 [17].

### 4.4. Comparison of Constitutive Relationships for Creep-Recovery Properties with Test Results

Creep strain analysis was carried out under cyclic loadings, taking into account the integral hereditary law for creep strains (14) and the following three long-term standard models: the EC2:2004 [66] standard model, the MC 2010 pre-standard [15] model, and the MC 2020 pre-standard [17] model. The total strains were measured for both the LC1 and LC2 concrete mixes assuming the following loading program (see Section 2.4 and Section 3.4):

- For concrete made from the LC1 mix, the first loading phase lasted for 419 days at a stress of 15.55 MPa; the first unloading phase at a stress of 1.56 MPa lasted until 572 days after the first load was applied; the second loading phase at 15.55 MPa lasted until 724 days; the second unloading phase at 1.56 MPa lasted until 897 days; and the third loading phase at 15.55 MPa lasted until 1050 days after the first load was applied.
- For concrete made from the LC2 mix, the first loading phase lasted for 413 days at a stress of 16.96 MPa; the first unloading phase at a stress of 1.70 MPa lasted until 566 days after the first load was applied; the second loading phase at 16.96 MPa lasted until 718 days; the second unloading phase at 1.70 MPa lasted until 891 days; and the third loading phase at 16.96 MPa lasted until 1044 days after the first load was applied.

The data given below were used to calculate strain values according to the individual long-term models and were applied at those times when the load on the samples changed. For the LC1 concrete samples, these times were: *t*_0_ = 28 days, *t*_1_ = 419 + 28 = 447 days, *t*_2_ = 572 + 28 = 600 days, *t*_3_ = 724 + 28 = 752 days, *t*_4_ = 897 + 28 = 925 days. For the LC2 concrete samples, these times were: *t*_0_ = 28 days, *t*_1_ = 413 + 28 = 441 days, *t*_2_ = 566 + 28 = 594 days, *t*_3_ = 718 + 28 = 746 days, *t*_4_ = 891 + 28 = 919 days.

The development of the elasticity modulus of concrete, for the model according to EC2:2004 [66], is described by Formula (2) and Equation (3.2) in EC2:2004 [66]; however, in the comparative calculations carried out here, due to the phenomenon of reduction in the tensile strength and the value of the elasticity modulus of the considered lightweight concrete resulting from the development of shrinkage microcracks after completing the curing of the concrete (after 28 days), the value of the secant modulus of elasticity of concrete *E_cm_* at the age of 28 days, as assumed for analysis, was modified by introducing the so-called adjusted modulus of elasticity. The basis was taken as the experimentally obtained values of the secant modulus of elasticity of concrete aged approximately one year, which were determined as average values from 3 samples and amounted to approximately 23.62 GPa for LC1 concrete and 23.87 GPa for LC2 concrete, respectively. These translate into the values of the adjusted secant elastic modulus of concrete *E_cm_* at the age of 28 days: 22.35 GPa for LC1 concrete and 22.59 GPa for LC2 concrete, respectively. By substituting these *E_cm_* values into Formula (2) for the evolution of the secant modulus of elasticity, and *s* = 0.25 and *n* = 0.3 into Equation (3.2) of EC2:2004 [66], and assuming *t* = 400 days at the time of testing the samples, one can again obtain the above-mentioned values of the secant modulus of elasticity for LC1 and LC2 concrete aged approximately one year. A comparison of the development of the secant modulus of elasticity for concrete samples LC1 and LC2 according to the tests and the EC2:2004 [66] model is shown in Figure 4 and Figure 5. The tangent modulus *E_c_*(*t*) is determined by Formula (1). For the model according to the MC 2010 pre-standard [15], the data were taken as for the EC2:2004 [66] model, assuming, however, that *n* = 0.5. For the model according to the MC 2020 pre-standard [17], the data were also taken as for the EC2:2004 [66] model, assuming in this case *n* = 1/3. These assumptions influenced the values of the adjusted secant elastic modulus of concrete *E_cm_* at the age of 28 days, which for MC 2010 [15] amounted to 21.54 GPa for LC1 concrete and 21.77 GPa for LC2 concrete, respectively, and for the model according to MC 2020 [17] amounted to 22.22 GPa for LC1 concrete and 22.45 GPa for LC2 concrete, respectively. Substituting the above *E_cm_* values into Formula (2) and assuming *s* = 0.25 and *n* = 0.5 for the model according to MC 2010 [15], or *n* = 1/3 for the model according to MC 2020 [17], and assuming *t* = 400 days (at the time of testing the samples), the experimentally obtained values of the secant modulus of elasticity of LC1 and LC2 concretes aged approximately one year can be obtained again, i.e., approximately 23.62 GPa for LC1 concrete and 23.87 GPa for LC2 concrete. A comparison of the development of the secant modulus of elasticity of concrete samples LC1 and LC2 according to the tests and both models from the pre-standards MC 2010 [15] and MC 2020 [17] is also shown in Figure 4 and Figure 5, respectively. For the models according to both pre-standards, the tangent modulus *E_c_*(*t*) is determined by Formula (1).

The development of concrete shrinkage, for the model according to EC2:2004 [66], is described by the system of formulas from (3) to (11), which were used in the calculations. However, the comparative analysis carried out herein considered lightweight concrete, and, based on experimental tests, it was assumed that the strength class of concrete for both the LC1 and LC2 mixtures is LC 45/50. In addition, the coefficient values *α_ds_*_1_ = 4.00 and *α_ds_*_2_ = 0.12 were assumed for the selected cement class N; the relative humidity of the environment was assumed as *RH* = 50%, in accordance with the results of humidity measurements in the measuring chamber; and the nominal cross-sectional dimension was *h*_0_ = 47 mm, hence *k_h_* = 1.0. A comparison of the shrinkage development for concrete samples LC1 and LC2 according to the standard tests [90] and the EC2:2004 [66] model (for the beginning of drying *t*_s_ = 21 days) is shown in Figure 7. For the model according to the MC 2010 [15], the data was taken as for the model according to EC2:2004 [66], but with *α_ds_*_2_ = 0.012 due to differences in the structure of Formulas (B.11) in EC2:2004 [66] and (8) (Section 4.2). For the model according to MC 2020 [17], the data were taken as for the model according to MC 2010 [15] model. A comparison of the shrinkage development for concrete samples LC1 and LC2 according to the standard tests [90] and the EC2:2023 [67] model (for the beginning of drying *t*_s_ = 21 days) is shown in Figure 7.

The description of concrete shrinkage development for the models based on the pre-standards MC 2010 [15] and MC 2020 [17] contains analogous forms of the coefficients necessary to determine shrinkage strains as in the model according to EC2:2023 [67]; however, when determining the model coefficients, only the appropriate signs in the formulas should be considered. For this reason, the descriptions of concrete shrinkage development based on the above documents are not distinguished in the present study.

The development of concrete creep strains, for the EC2:2004 [66] model, is described by Formulas (28) to (32) with the reduction factor *η_E_* = 0.67, which were used in the calculations. It should be noted that the comparative analysis carried out here considers lightweight concrete and assumes that the strength of LC1 and LC2 concrete is in both cases of the LC 45/50 class; the ambient relative humidity was assumed to be *RH* = 50%, according to the results of humidity measurements in the measuring chamber; the nominal cross-sectional dimension was *h*_0_ = 47 mm; and the value of the coefficient *α* = 0 was assumed in Expression (B.9) in EC2:2004 [66] for the selected cement class N.

The creep curve for the initial load in the form of hereditary strains is present in the mathematical description until the end of the cyclic loading (see, e.g., Equation (25), first part). When comparing the results of the of creep strain tests for LC1 concrete with the results of calculations according to EC2:2004 [66], the resulting correction factor (at *η_E_* = 0.67 as above) was applied to this curve, determined by the least squares error method, amounting to *η_E_ξ_c_* = 1.28. For LC2 concrete, the resulting factor *η_E_ξ_c_* = 1.24, determined in the same way, was applied. This ensured consistency between the results for the EC2:2004 [66] model and the LC1 concrete test results for the loading period from 0 to 419 days, and between the EC2:2004 [66] model results and the LC2 concrete test results for the loading period from 0 to 413 days. However, for creep strains due to unloading and reloading occurring after a period of 419 and 413 days for both concretes, respectively, only the correction factor *η_E_* was applied, because neither the MC 2020 pre-standard [17] nor the EC2:2023 standard [67] specifies other factors for cyclic loads in this case. A comparison of the creep-recovery strains of concrete samples LC1 and LC2 according to the tests and the EC2:2004 [66] standard model is shown in Figure 8 and Figure 10, respectively, and the comparison of the total strains of both concrete samples according to the tests and the considered model is shown in Figure 9 and Figure 11, respectively.

According to the MC 2010 pre-standard [15] (Formulas (33) to (46), which were also used in the calculations), the data and correction factor *η_E_* = 0.67 were assumed as for the EC2:2004 [66] model. For the model according to the MC 2020 pre-standard [17] (Formulas (47) to (49)), the data were also assumed as for MC 2010 [15], however, with *α_SC_* = 0 (instead of *α* = 0 in Formula (46)), due to for differences in the structure of the formulas determining the adjusted time *t*_0,*adj*_. When comparing the results of creep strain tests for LC1 concrete with the results for the MC 2010 [15] model for the creep curve under initial load, the resulting correction factor determined by the least squares method, *η_E_ξ_c_* = 1.38, was used. For LC2 concrete and the MC 2010 [15] model, the correction factor determined in the same way, *η_E_ξ_c_* = 1.30, was applied, which ensured agreement between the results for the MC 2010 [15] model and the test results for the loading period from 0 to 419 days (LC1 concrete) or from 0 to 413 days (LC2 concrete). For unloading and reloading occurring after a period of 419 and 413 days for both concretes, respectively, only the correction factor *η_E_* was applied.

**Figure 12:**
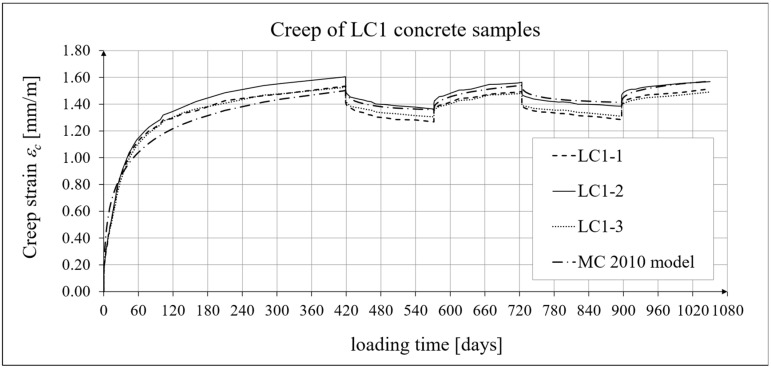
Comparison of creep-recovery strain according to MC 2010 [15] model and test results of LC1 concrete samples.

**Figure 13:**
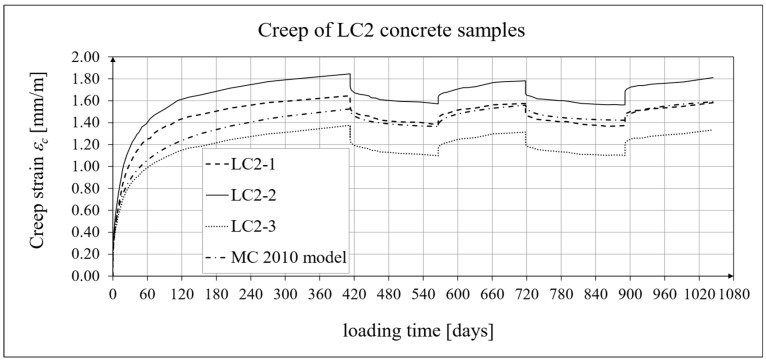
Comparison of creep-recovery strain according to MC 2010 [15] model and test results of LC2 concrete samples.

**Figure 14:**
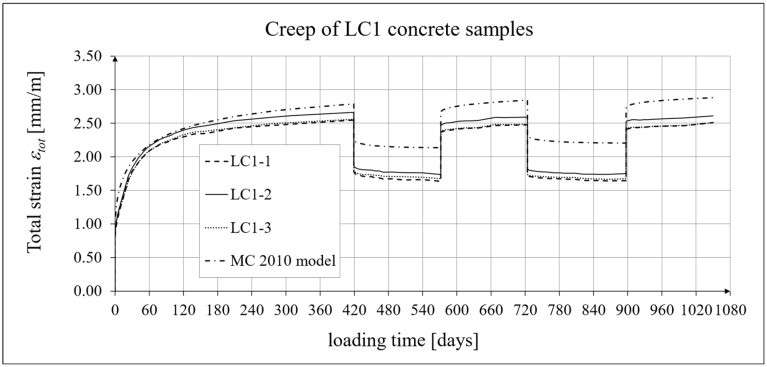
Comparison of total strain according to MC 2010 [15] model and test results of LC1 concrete samples.

**Figure 15:**
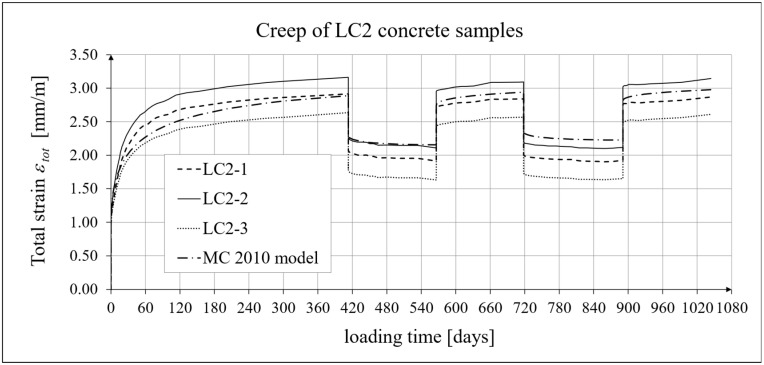
Comparison of total strain according to MC 2010 [15] model and test results of LC2 concrete samples.

**Figure 16:**
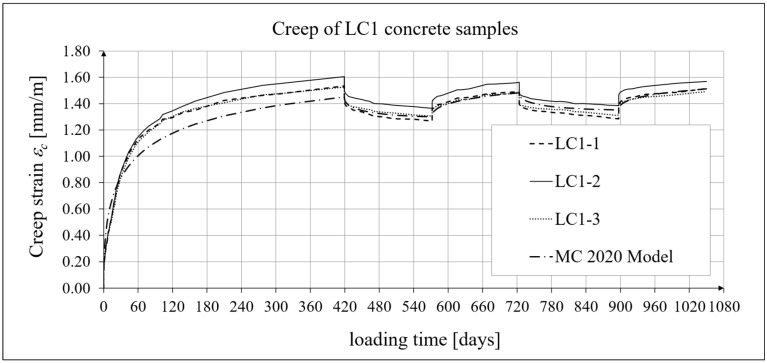
Comparison of creep-recovery strain according to MC 2020 [17] model and test results of LC1 concrete samples.

**Figure 17:**
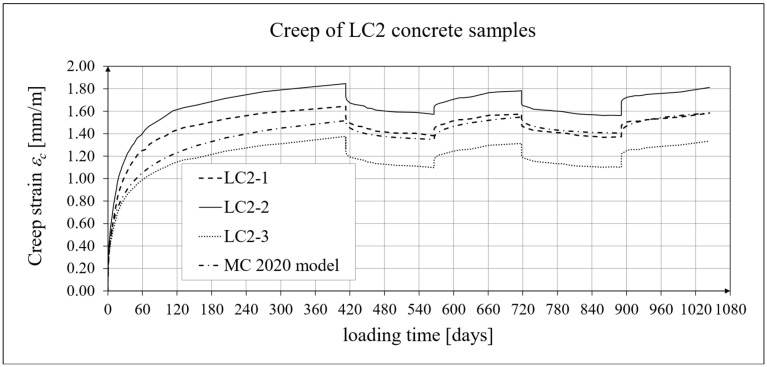
Comparison of creep-recovery strain according to MC 2020 [17] model and test results of LC2 concrete samples.

**Figure 18:**
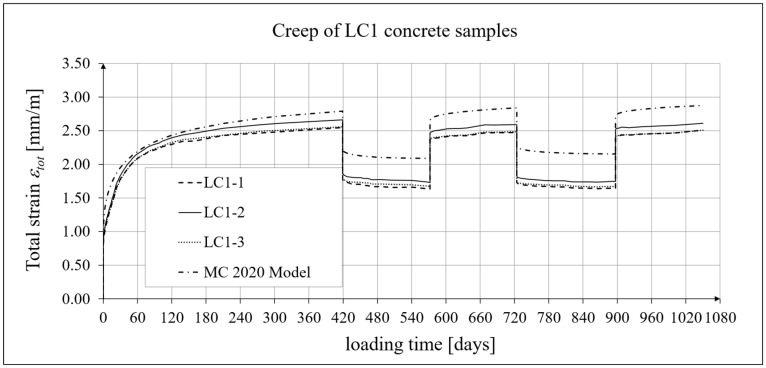
Comparison of total strain according to MC 2020 [17] model and test results of LC1 concrete samples.

**Figure 19:**
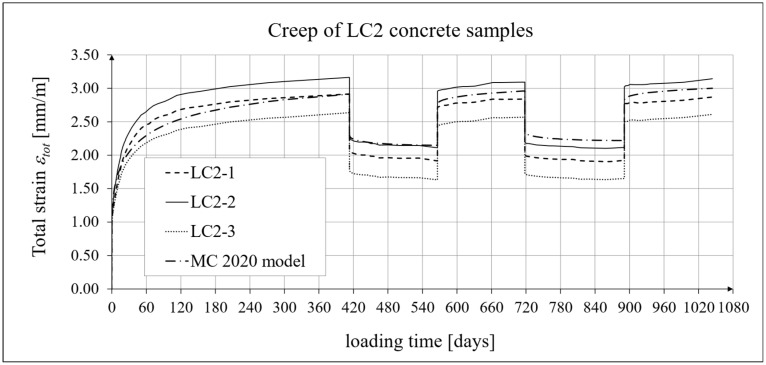
Comparison of total strain according to MC 2020 [17] model and test results of LC2 concrete samples.

A comparison of the creep behavior of concrete samples LC1 and LC2 according to the tests and the MC 2010 pre-standard [15] model is presented in Figure 12 and Figure 13, respectively, and the comparison of the total strains of the samples of both concretes according to the tests and the MC 2010 [15] model is presented, respectively, in Figure 14 and Figure 15. Similarly, when comparing the results of creep strain tests for LC1 concrete with the results for the MC 2020 [17] model for the creep curve under initial load, the resulting correction factor determined by the least squares method, *η_E_ξ_c_* = 1.28 was used. For LC2 concrete and the MC 2020 [17] model, the correction factor determined in the same way, *η_E_ξ_c_* = 1.24, was applied, which ensured agreement between the results for the MC 2020 [17] model and the test results for the loading period from 0 to 419 days (LC1 concrete) or from 0 to 413 days (LC2 concrete). For load increments occurring after a period of 419 and 413 days for both concretes, respectively, only the correction factor *η_E_* was applied. A comparison of the creep behavior of concrete samples LC1 and LC2 according to the tests and the MC 2020 pre-standard [17] model is presented in Figure 16 and Figure 17, respectively, and the comparison of the total strains of the samples of both concretes according to the tests and the MC 2020 [17] model is presented, respectively, in Figure 18 and Figure 19.

Comparing the results of the total strain and creep strain analyses obtained using the EC2:2004 [66] standard model with the results obtained using the models according to the pre-standards MC 2010 [15] and MC 2020 [17] (see the graphs in Figure 12, Figure 13, Figure 14, Figure 15, Figure 16, Figure 17, Figure 18 and Figure 19 compared with the graphs in Figure 8, Figure 9, Figure 10 and Figure 11), it can be concluded that the modifications introduced in the pre-standard models [15,17] compared to the model according to EC2:2004 [66] do not improve the approximation of the test results for the type of lightweight concrete considered.

## 5. Discussion

Samples of lightweight concrete with sintered ash-derived aggregate, prepared with two W/C ratios, 0.4 for the LC1 mix and 0.5 for the LC2 mix, were subjected to cyclic loading. The tests enabled the experimental determination of the long-term properties of this concrete in terms of changes in its mechanical parameters under the loads described in this paper, as well as the development of appropriately adapted mathematical models consistent with the general principles outlined in the latest standards and pre-standards. The test results presented in this paper include readings from the entire research process, which spanned over 1000 days after the samples were prepared. The graphs of the cubic and cylindrical compressive strength of lightweight concrete were prepared for the first 300 days of concrete age, illustrating the development of concrete strength for both tested mixture types over time (see Figure 3).

For the secant modulus of elasticity test results of for concretes from both the LC1 and LC2 mixtures, as well as the standard empirical relationships for this modulus, the graphs were prepared for the first 700 days of concrete age (see Figure 4 and Figure 5).

The shrinkage strain tests using the Amsler method [89] were carried out at the intervals specified in Section 2.4, and the graphs were prepared for the first 325 days of concrete age (see Figure 6). Shrinkage strain tests using the standard method [90] were also carried out at the intervals specified in Section 2.4, but the graphs were prepared only for the first 510 days of concrete age (see Figure 7), although the entire process of recording shrinkage strains lasted 1050 days for the LC1 mix and 1044 days for the LC2 mix. Due to the stabilization of the test results, the graphs are limited to 510 days. At the same time intervals as the shrinkage strain tests conducted according to standard [90], the cyclic total strain and creep strain were measured at the intervals also described in Section 2.4. These tests also lasted 1050 days for the samples from the LC1 mixture and 1044 days for the samples from the LC2 mixture. Graphs showing the cyclic creep strain and total strain for specimens made from both concrete mixtures, LC1 and LC2, are shown in Figure 8, Figure 9, Figure 10, Figure 11, Figure 12, Figure 13, Figure 14, Figure 15, Figure 16, Figure 17, Figure 18 and Figure 19.

Regarding parameterization and modeling of constitutive equations, an analysis of creep deformations under cyclic loads was carried out, taking into account the integral hereditary law (14) and three long-term models formulated according to the following standards and pre-standards: EC2:2004 [66], MC 2010 [15], and MC 2020 [17], which is compliant with the EC2:2023 standard [67]. The results from the individual models were compared with the test results, and the process of generalizing the models to account for cyclic loads was based on the general provisions of the MC 2020 pre-standard [17], using the integral hereditary law (also presented in this pre-standard) according to the Boltzmann superposition principle and the rules for determining correction factors for models.

The difficulty in modeling the secant modulus of elasticity of concrete stems from the specific properties of lightweight aggregate concrete, assuming a 28-day curing period. Standard models do not provide a description of the phenomena that may occur in this context. After 28 days, shrinkage strains had not yet reached half of their final value, while drying shrinkage still occurred at the interface between the cement matrix and the sintered aggregate, which was significantly greater than at the interface with natural aggregate. Consequently, microcracks may have formed, reducing the stiffness of the cement matrix sufficiently to affect the modulus of elasticity of the concrete with sintered aggregate. Due to the possibility of shrinkage microcracks occurring after the concrete curing process, the value of the secant modulus of elasticity of concrete *E_cm_* at the age of 28 days, adopted for analysis, was modified based on experimental data by introducing a corrected modulus of elasticity.

Similarly, there were differences in the assessment of shrinkage and creep strains of lightweight concrete with sintered aggregate compared to the model predictions specified in the standards and pre-standards. However, the latest versions of these documents require the determination of appropriate correction factors for both shrinkage and creep strains. Regarding the concrete deformation resulting from creep, the tests showed that the average final creep coefficient (after more than one year) was approximately 2.1 for the LC1 concrete samples and approximately 1.9 for the LC2 concrete samples (differing from the standard model predictions [66,67]); therefore, the standard model curves had to be adjusted accordingly. The average creep coefficient obtained in work [77] for both concretes ranged from approximately 0.59 to 0.60, which differs from both the results of these tests and the predictions of the code models [66,67].

Comparing the calculated shrinkage strain development values according to the EC2:2004 [66] model, assuming a drying shrinkage coefficient *η*_3_ = 1.2, as shown in Figure 6, and the shrinkage strain values calculated according to the EC2:2023 [67] model, assuming a total shrinkage coefficient *η* = 1.2, as also shown in Figure 6, with the experimental results for the shrinkage strain development of LC1 and LC2 concrete samples, obtained using the Amsler method [89], it can be concluded that the EC2:2023 [67] model provides a better fit to the experimental results for the first 325 days of concrete age.

The use of a correction factor, in this case *η_E_* = 0.67, pertaining to the creep of LWAC in accordance with the requirements of EC2:2004 [66] and EC2:2023 [67], is more controversial. While the use of porous fly ash aggregate undoubtedly increases shrinkage strains, the resulting reduction in creep is not obvious, because the cement matrix plays the primary role in the creep phenomenon. The average final creep coefficient values reported above (after more than one year) do not indicate a reduction in creep for LWAC. However, the creep-recovery strain values during subsequent unloading and reloading intervals are much lower, and in this respect, it would be reasonable to retain the reduction factor. Since the latest standard EC2:2023 [67] and the MC 2020 pre-standard [17] allow for the use of empirically determined correction factors, such factors can be determined for the initial load, thus achieving consistency with the test results. Therefore, in the case of cyclic loading, the use of such a reduction factor *η_E_* is justified.

The development of creep deformations is correctly modeled by the standard methods (EC2:2004 [66], MC 2010 [15], MC 2020 [17]), albeit with appropriate correction factors that should be determined so as to minimize the sum of the squares of the differences between the model estimation and the experimental results (see MC 2020 [17]). However, when comparing the results of the analyses of total deformations and creep deformations obtained using the standard Model 5 according to EC2:2004 [66] with the results obtained using Models 6 and 7 according to the pre-standards MC 2010 [15] and MC 2020 [17] (cf. graphs in Figure 12, Figure 13, Figure 14, Figure 15, Figure 16, Figure 17, Figure 18 and Figure 19 with graphs in Figure 8, Figure 9, Figure 10 and Figure 11), it can be concluded that the modifications introduced in Model 5 do not improve the approximation of the test results for the concrete type under consideration. Regarding the test and analysis results for shrinkage strains over a longer period of more than 500 days (see graphs in Figure 7), the corrections introduced in the EC2:2023 standard [67] model still do not reflect the influence of the W/C ratio.

Cyclic stress changes did not increase the creep strains. One of the research objectives was to determine whether the ratchetting phenomenon could be observed during creep of the concrete under consideration subjected to cyclic loading; however, due to the very low level of plastic deformation, this phenomenon was not detected.

Comparing the creep strains at the end of the first loading interval and the first unloading interval, the average recovery magnitude for LC1 concrete was found to be 15.62%, and the average recovery magnitude for LC2 concrete was 16.75%. A comparison of this effect was made with the test results for LWAC mixtures C-1 and C-2 described in [77], which are similar to the LC1 and LC2 mixtures, as they were developed based on the same LSA recommendations. To determine the recovery rate (percentage of original creep), Figure 10 [77] was used. This figure refers to strains defined as “the difference between strains of loaded and unloaded specimens”, meaning the difference between the total strains and the shrinkage strains, and therefore the sum of the elastic and creep strains. The strains of 322 and 365, respectively, for mixtures C-1 and C-2, shown in Figure 10, refer to the “instantaneous strains” [77]. Thus, the creep strains at the end of the first loading interval are 187 and 248 for mixtures C-1 and C-2, respectively. The calculations show that the average recovery value for C-1 and C-2 concrete is 16.44%, while according to our research, the average recovery value for LC1 and LC2 concrete is 16.19%; thus, it is of the same order of magnitude.

Moreover, the analysis for cyclic loads shows that correcting the Boltzmann–Volterra superposition principle (14) to the form defined by Formula (15) is unnecessary (provided the initial condition is correctly formulated), although such a corrected equation is cited in many documents, including MC 2010 [15] and MC 2020 [17]. For this reason, the expressions in the form of integral Equations (15) and (17) prove to be redundant. However, since the integral equation of the hereditary law in the form of (15) is cited in many documents, including the MC 2010 [15] and MC 2020 [17], the integral equations of the form (15) and (17) may be simplified.

## 6. Conclusions

Based on the tests and analyses conducted, the following conclusions can be drawn.

### 6.1. Conclusions for Experimental Results

- The average final creep coefficient values (after at least one year) of lightweight concrete with the examined sintered aggregate were similar of those of plain concrete of the same strength.
- Due to the low plastic strain of LWAC, a relatively low recovery magnitude (percentage of original creep) was observed compared to plain concrete.
- A limitation of the study was restricted access to creep testing machines—only two machines were available (although the ITB laboratory is equipped with more such devices), which limited the number of samples. Consequently, three samples were used to test the creep of LC1 concrete, and three samples were also used to test the creep of LC2 concrete.
- A limitation of the study was the relatively large scatter in the shrinkage results for the LC2 concrete mix, tested in accordance with the European standard, which was caused by variations in the moisture content of the aggregate used in the production of this concrete mix (the mix production took place outside the ITB laboratory).
- The stress changes did not increase the creep strains of the concrete under consideration subjected to cyclic loading, and the ratchetting phenomenon, attributable to very low plastic strain levels, was not detected.
- Further research is planned on concrete mixtures with lightweight aggregate containing additives that improve the concrete’s tensile strength, modulus of elasticity and frost resistance.

### 6.2. Conclusions for Model Calibration Results

- The EC2:2023 [67] shrinkage strain model provides a better match to the experimental results for the first 325 days of concrete age compared to the EC2:2004 [66] model.
- The development of creep-recovery strain for the examined LWAC is correctly modeled using the EC2:2004 [66] or EC2:2023 standard [67] models and the integral equations of the hereditary law, also provided in EC2:2023 [67] and MC 2020 [17], albeit with appropriate correction factors. In accordance with the requirements of EC2:2023 [67], these factors must be determined by minimizing the sum of squares of the differences between the model estimates and the experimental results. The necessity of using these factors results from the sensitivity of LWAC to the method of preparing the porous aggregate before concreting.
- Comparing the results of the analyses of total strain and creep strain obtained using the standard models, it can be concluded that the modifications introduced in the EC2:2023 [67] model compared to the EC2:2004 [66] model do not improve the approximation of the test results for the concrete type under consideration.
- Correcting the Boltzmann–Volterra superposition principle in its basic form (14) is unnecessary (provided the initial condition is properly formulated).

### 6.3. Direct Implications for Design Engineers

- The description of LWAC creep and shrinkage requires the use of the correction factors provided for in the EC2:2023 standard [67].
- Since the ratchetting phenomenon was not detected, the type of LWAC discussed can be used in building structures subjected to cyclically varying loads with long loading intervals.
- The research described in this paper confirmed the suitability of lightweight concrete with sintered ceramic aggregate derived from fly ash for use in cyclically loaded prestressed structures.

## Figures and Tables

**Table 1:** Concrete mixtures based on LSA recommendations (see article [1]).

Component	LC1 Mix	LC2 Mix
	Dosage [kg/m^3^]	
Cement CEM I 42.5 N	409	419
Lightweight sintered aggregate Certyd 4/10	775	802
Sand	682	703
Water	164	209
Admixture BV 18	3.7	3.8
Admixture SKY 686	3.7	3.8

**Table 2:** Summary of LC1 test results with measurement uncertainty *U* for *t* = 419 days.

Sample No.	*ε_tot_* ± *U*	*ε_e_* ± *U*	*ε_c_* ± *U*	*φ* ± *U*
	[mm/m]	[mm/m]	[mm/m]	[mm/m]
LC1-1	2.55 ± 0.07	0.72 ± 0.04	1.54 ± 0.07	2.14 ± 0.07
LC1-2	2.66 ± 0.07	0.76 ± 0.04	1.61 ± 0.07	2.12 ± 0.07
LC1-3	2.56 ± 0.07	0.74 ± 0.04	1.53 ± 0.07	2.07 ± 0.07
average value	2.59	0.74	1.56	2.11
standard deviation	0.061	0.020	0.044	0.037

**Table 3:** Summary of LC2 test results with measurement uncertainty *U* for *t* = 413 days.

Sample No.	*ε_tot_* ± *U*	*ε_e_* ± *U*	*ε_c_* ± *U*	*φ* ± *U*
	[mm/m]	[mm/m]	[mm/m]	[mm/m]
LC2-1	2.92 ± 0.07	0.82 ± 0.04	1.65 ± 0.07	2.01 ± 0.14
LC2-2	3.16 ± 0.08	0.87 ± 0.04	1.84 ± 0.07	2.11 ± 0.14
LC2-3	2.64 ± 0.07	0.81 ± 0.04	1.38 ± 0.07	1.70 ± 0.13
average value	2.91	0.83	1.62	1.94
standard deviation	0.260	0.032	0.231	0.214

**Table 4:** The expanded measurement uncertainty (*U* [%]) of LC1 concrete test results for *t* = 419 days and LC2 concrete results for *t* = 413.

Sample No.	*U* (*ε_tot_*)	*U* (*ε_e_*)	*U* (*ε_c_*)	*U* (*φ*)
	[%]	[%]	[%]	[%]
LC1-1	2.75	5.56	4.55	8.42
LC1-2	2.63	5.26	4.35	8.02
LC1-3	2.73	5.41	4.58	8.22
LC2-1	2.40	4.88	4.24	6.96
LC2-2	2.53	4.60	3.80	6.62
LC2-3	2.65	4.94	5.07	7.63

## Data Availability

The original contributions presented in the study are included in the article. Further inquiries can be directed to the corresponding author.

## Abbreviations

The following abbreviations are used in this manuscript:

ITB	Instytut Techniki Budowlanej (Building Research Institute)
LSA	Lightweight Sintered Aggregate
LWAC	Lightweight Aggregate Concrete
IMiKB	Instytut Materiałów i Konstrukcji Budowlanych (Inst. of Building Mater. & Struct.)
W/C	Water/Cement Ratio
LC	Lightweight Concrete
CEM	Cement Class
CEB-FIP	the merger of CEB and FIP
CEB	Euro-International Committee for Concrete
FIP	International Federation for Prestressing
*fib*	International Federation for Concrete (the merger of CEB and FIP)
MC 2010	Model Code 2010
MC 2020	Model Code 2020
EC2:2004	EN 1992-1-1:2004 Eurocode 2
EC2:2023	EN 1992-1-1:2023 Eurocode 2
